# Embracing the positive: an examination of how well resilience factors at age 14 can predict distress at age 17

**DOI:** 10.1038/s41398-020-00944-w

**Published:** 2020-08-05

**Authors:** J. Fritz, J. Stochl, I. M. Goodyer, A.-L. van Harmelen, P. O. Wilkinson

**Affiliations:** 1 Department of Psychiatry, University of Cambridge, Cambridge, UK; 2 Department of Kinanthropology, Charles University, Charles, Czech Republic

**Keywords:** Psychiatric disorders, Diseases

## Abstract

One-in-two people suffering from mental health problems develop such distress before or during adolescence. Research has shown that distress can predict itself well over time. Yet, little is known about how well resilience factors (RFs), i.e. those factors that decrease mental health problems, predict subsequent distress. Therefore, we investigated which RFs are the best indicators for subsequent distress and with what accuracy RFs predict subsequent distress. We examined three interpersonal (e.g. friendships) and seven intrapersonal RFs (e.g. self-esteem) and distress in 1130 adolescents, at age 14 and 17. We estimated the RFs and a continuous distress-index using factor analyses, and ordinal distress-classes using factor mixture models. We then examined how well age-14 RFs and age-14 distress predict age-17 distress, using stepwise linear regressions, relative importance analyses, as well as ordinal and linear prediction models. Low brooding, low negative and high positive self-esteem RFs were the most important indicators for age-17 distress. RFs and age-14 distress predicted age-17 distress similarly. The accuracy was acceptable for ordinal (low/moderate/high age-17 distress-classes: 62–64%), but low for linear models (37–41%). Crucially, the accuracy remained similar when only self-esteem and brooding RFs were used instead of all ten RFs (ordinal = 62%; linear = 37%); correctly predicting for about two-in-three adolescents whether they have low, moderate or high distress 3 years later. RFs, and particularly brooding and self-esteem, seem to predict subsequent distress similarly well as distress can predict itself. As assessing brooding and self-esteem can be strength-focussed and is time-efficient, those RFs may be promising for risk-detection and translational intervention research.

## Introduction

Every year, about 1 in 5 people experience mental disorders^1,2^, of which the most prevalent mental illnesses are depressive and anxiety disorders^1^. Half of such mental illnesses first emerge during adolescence^3^. About 1 in 3 adolescents have an episode of an anxiety disorder and more than 1 in 10 an episode of a mood disorder, between the ages of 13 and 18^4^. The prevalence of anxiety disorders tends to remain stable during adolescence, however, mood disorders double between the ages of 13 and 18^4^. Hence, adolescence seems to be a particularly sensitive time period for the emergence of mental health problems and it is therefore imperative to characterise and predict such vulnerability to psychopathological distress properly.

A growing number of studies has developed screening tools and risk prediction models—also known as risk calculators—for mental health problems^5,6^. For example, Dinga et al.^7^ have shown that, among a large variety of psychological and biological variables, only mood severity predicted subsequent depressive symptomology significantly^7^. Still, their prediction model revealed an acceptable accuracy^7^. Similarly, Lewis et al. have shown that a constellation of demographics, psychopathology symptoms (i.e. psychotic and internalising symptoms), and adversity variables can together satisfactorily predict whether adolescents develop post-traumatic stress disorder, following trauma exposure^8^. In a recent systematic review, summarising literature on mental health screening tools and risk models, 60 studies were identified for depression related diagnoses, 13 for psychopathological stress, five for anxiety related diagnoses, and five for well-being^6^. Importantly, the majority of those studies used symptom-related (e.g. questionnaires and interviews), demographical (e.g. adverse life-events), or biological indicators (e.g. inflammatory markers, cortisol, metabolic syndrome, brain-derived neurotrophic factor, white and grey matter, and heart rate variables)^5–7,9^. Thus, previous studies primarily examined predictors that are relatively static (e.g. ethnicity or grey matter) and/or risk factors that increase the development of mental health problems (e.g. negative life events or prior psychiatric symptoms).

Focussing on static and risk factors, however, is only half the story, as it fails to address factors that are amenable and promote mental health. The resilience literature has already identified various factors that are associated with improved subsequent mental health^10–14^, which seem to be overlooked in the development of screening tools and risk calculators. A notable exception is the study of Chen et al.^15^ in which self-esteem was used to predict subsequent anxiety. Another important exception is the recent study of Meehan et al.^16^, which included alongside various risk indicators four potential resilience factors (sibling warmth, adult involvement, social cohesion, and status among peers), to predict internalising and externalising disorders following victimisation.

Here, we aim to extend the existing prediction literature in several ways. Firstly, we use resilience factors (RFs) as predictors, i.e. factors that have been found to reduce the risk of psychological distress following adverse experiences^17^. We derived the RFs that we study here from a preregistered systematic review^17^, in which RFs were defined as those factors that moderate and/or mediate the relationship between childhood adversity and subsequent mental health problems. In the resilience literature there is a sparse but ongoing discourse about whether resilience and risk factors are opposing sides of the same coin (for a detailed discussion see Fritz et al.^18^). Some RFs and risk factors seem indeed to be on opposing sides of the same continuum (e.g. RF = high friendship support, and risk factor = low friendship support)^19^, whereas for others this apparent dichotomy seems more complex. For example, *high* rumination can be both an RF and a risk factor depending whether its content is positive or negative (e.g. RF = *high positive* rumination, and risk factor = *low positive* ruminations; RF = *low negative* rumination, and risk factor = *high negative* rumination; while *high positive* and *high negative* rumination often go together^20^). Importantly, regardless of whether resilience and risk factors operate on the same continuum, studying the predictive value of RFs has universal appeal as it focuses on what promotes good mental health rather than on what increases mental health problems^18^.

Secondly, we extend the existing literature through focusing exclusively on factors that are amenable to psychotherapeutic change, which is in contrast to the majority of the above reviewed studies, as those mainly focused on relatively static demographic (e.g. ethnicity) and biological (e.g. grey or white matter volume) predictors. More specifically, we predict psychopathological distress from ten amenable RFs. Three of those RFs operate on an *inter*-individual level: friendship support, family support and family cohesion; and seven on an *intra*-individual level: high positive self-esteem, low negative self-esteem, low brooding, low ruminative reflection, high distress tolerance, a low aggression potential and low expressive suppression^17^. Importantly, all those RFs on their own have been found to decrease subsequent mental health problems, yet, research investigating multiple RFs at the same time is so far scarce^21–23^. Recently, we found that these RFs reduce concurrent psychopathological distress with a similar degree in adolescents with and without prior exposure to adversity. Moreover, we have shown that the RFs interrelate strongly and can be described as a complex interacting system^18^. This supports the notion that models that succeed in taking all those factors into account may ecologically be more valid and may successfully reveal those RFs that are particularly important in reducing the risk of mental health problems.

Recently, research has also shed light on the benefits of describing mental health problems as distress continua rather than as discrete diagnosis specific constructs. For example, several studies show that modelling psychopathological symptoms as a continuous latent factor captures a wide range of mental health symptomatology, in terms of both severity and breadth of symptomatology^24–28^ and even seems to generalise well to other disorders^25^. Therefore, such latent continuous constructs may be particularly informative for transdiagnostic prevention and intervention research. Moreover, hybrid models have been developed that describe mental health symptoms as a continuous latent factor and then add categorical classes to the latent factor that differentiate between subgroups on the latent mental distress continuum (e.g. as defined by differences in the distress severity)^29^. Categorical distress scores derived from those models may be particular useful for prediction purposes, as they allow for the estimation of predictive sensitivity and specificity, while taking into account the continuous nature of distress. Yet, to the best of our knowledge, transdiagnostic distress indices have so far rarely been used for predictive purposes and is therefore the third way in which we extend the existing literature.

In sum, we aim to extend the existing literature (a) by using RFs rather than risk markers as predictors for subsequent psychopathology, (b) by using amenable (i.e. social, emotional, cognitive and behavioural) rather than static variables (e.g. ethnicity or biological predispositions) as predictors, and (c) by using transdiagnostic distress indices rather than discrete diagnosis specific variables as outcome variables. To this end, we use data from the ROOTS population cohort (*n* = 1130)^30^ to predict distress at age 17 from RFs assessed at age 14, covering the adolescent period during which about half of all mental illnesses start emerging. Given the powerful predictive effects of past mental distress, we evaluate in addition to the relative effects of RFs also the relative effect of distress at age 14 when predicting distress at age 17. A cascade of studies has shown that childhood adversity (CA) vastly increases the risk for mental health problems during adolescence and adulthood^31–34^. Therefore, throughout all analyses, we take the effect of CA before the age of 14 into account. In addition, we control for gender effects, as being female has frequently been found to increase the risk for distress^26^. In sum, we aim to examine:

- to what degree RFs can explain subsequent distress,
- which RFs are the best indicators for subsequent distress, and
- with what accuracy RFs can predict distress levels 3 years later.

## Methods

### Sample

The ROOTS study is a population cohort for which 1238 adolescents were recruited at age 14 and reassessed at age 17. The adolescents were recruited in 2005 and 2006, via 18 schools in and around Cambridgeshire. The adolescents and one parent had to provide written informed consent. ROOTS was approved by the Cambridgeshire Research Ethics Committee (03/302) and was conducted along the lines of Good Clinical Practice guidelines and the Declaration of Helsinki^30^.

### Participants

Here we included all adolescents who had data for potential CA experiences (CA+: *n* = 638; CA−: *n* = 501) and had less than 85% missingness on the analyses variables (*n* = 1188). Prior to the main analyses we imputed missing data and could therefore eventually analyse data of 1130 adolescents, of which 631 with and 499 without prior exposure to CA, and of which 620 were female and 510 male.

### RFs

In accordance with Fritz et al.^23,18^, we investigated ten RFs that were identified in our preregistered systematic review^17^ and were assessed in ROOTS^30^. All RFs were assessed at age 14:

1. Friendship support: five items of the Cambridge Friendships Questionnaire^35^.
2. Family support: five items of the McMaster Family Assessment Device^36^.
3. Family cohesion/climate: seven items of the McMaster Family Assessment Device. For brevity we write family cohesion throughout the manuscript^36^.
4. Positive self-esteem: five items of the Rosenberg self-esteem scale^37^.
5. Negative self-esteem: five remaining items of the Rosenberg self-esteem scale (of note, the items are reversed)^37^.
6. Reflective rumination: five items of the Ruminative Response Scale (RRS; of note, the items are reversed)^38,39^.
7. Ruminative brooding: five items of the RRS (of note, the items are reversed)^38,39^.
8. Aggression: four items of the Behaviour Checklist (11 questions based on the DSM-IV criteria for conduct problems; of note, the items are reversed)^40,41^.
9. Distress tolerance: five items of the Emotionality Activity Sociability Temperament Survey^42^.
10. Expressive suppression*:* one item of the Antisocial Process Screening Device (of note, the item is reversed)^43^.

Items of five RFs had to be reversed to ensure that all RFs are scored in such a way that high values are protective. The first eight RFs are based on self-report, and the last two on parent report. The psychometrics of the RF measures are described in Additional file XIII in Fritz et al.^23^.

### Distress

At age 14 and 17, distress was assessed with 41 items of which 28 had a focus on anxiety symptoms (Revised Children’s Manifest Anxiety Scale^44^) and 13 a focus on depressive symptoms (Short Mood and Feelings Questionnaire^45^).

### Childhood adversity

CA was assessed with the Cambridge Early Experiences Interview, which is a semi-structured interview performed with the primary carer^46^. CAs were defined as adverse experiences or severely stressful events that happened between birth and the age of 14. The assessed CAs include a wide range of intra-family events/experiences (e.g. sexual, physical or emotional maltreatments, or parental mental illness), but also cover external events (e.g. a fire or exposure to war). For a detailed description see Dunn et al.^46^. These authors clustered the adolescents based on their CA experiences into four latent classes (i.e. no, moderate, severe and atypical parenting CA), separately for the time periods early (age 0–5), middle (age 5–11) and late childhood (age 11–14)^46^. As in previous reports on this sample^23^, we dichotomised the CA variable in CA+, which is ‘moderate, severe and/or atypical parenting CA’ for at least one of the three time periods, and CA−, which is ‘no CA’ for any of the three time periods.

### Analyses

#### Data imputation

Prior to the main analyses we imputed missing data. Most participants with missing data had missingness at age 17. Yet, some adolescents had missing data at age 14, and others had just incidentally missing items at age 14 and/or 17. Details can be found in Additional file IV of Fritz et al.^18^. Overall, missingness on the RFs and general distress could to some degree be explained by exposure to CA, gender, affective symptoms, and a prior psychiatric history (see Additional file IV Table 3 in Fritz et al.^18^). We used multivariate multiple imputation methods to estimate ten complete data sets with estimated scores for the missing data. For data with more than two categories we used predictive mean matching algorithms and for binary data logistic regression. To enhance the imputation model accuracy, we included 103 items measuring the RFs, 122 items measuring anxiety and depression symptoms, and seven explanatory variables (CA, gender, socio-economic status, prior psychiatric history, and age at occasion 1 and 2—for measurement details see Table 1), leading to a total of 232 items. We did not impute information for the CA factor as we believe that not all CA experiences are adequately predictable (e.g. a car crash). In sum, we were able to estimate data for 1188 participants.

**Table 1:** Sample description, split for CA and gender.

	CA+ (*n* = 631)	CA− (*n* = 499)	*χ*^2^/*z*/t	*p* value
Gender	Female = 358	Female = 262	1.85 (1)	0.17
	Male = 273	Male = 237		
Age 14^a^	14.49 (0.28)	14.48 (0.28)	−0.43 (1049.3)	0.67
Age 17^a^	17.49 (0.34)	17.48 (0.32)	−0.48 (1015.8)	0.63
SES	Hard pressed = 73	Hard pressed = 30	5.24	<0.001
	Moderate means = 36	Moderate means = 11		
	Comfortably off = 168	Comfortably off = 104		
	Urban prosperity = 37	Urban prosperity = 41		
	Wealthy achievers = 317	Wealthy achievers = 313		
Prior psychiatric	Yes = 199	Yes = 74	41.54 (1)	<0.001
History at age 14	No = 432	No = 425		
Prior psychiatric	Yes = 267	Yes = 122	48.05 (1)	<0.001
History at age 17^a^	No = 297	No = 345		

	Female (*n* = 620)	Male (*n* = 510)	*χ*^2^/z/t	*p* value
Age 14^a^	14.49 (0.27)	14.48 (0.29)	0.61 (1027)	0.54
Age 17^a^	17.50 (0.32)	17.47 (0.34)	1.38 (954.71)	0.17
SES	Hard pressed = 51	Hard pressed = 52	1.33	0.18
	Moderate means = 23	Moderate means = 24		
	Comfortably off = 154	Comfortably off = 118		
	Urban prosperity = 34	Urban prosperity = 44		
	Wealthy achievers = 358	Wealthy achievers = 272		
Prior psychiatric	Yes = 176	Yes = 97	12.90 (1)	<0.001
History at age 14	No = 444	No = 413		
Prior psychiatric	Yes = 249	Yes = 140	16.66 (1)	<0.001
History at age 17^a^	No = 326	No = 316		

For age we depict the mean values and the belonging standard deviations in brackets. The Pearson’s *χ*^2^ tests were used for binary data and performed with Yate’s continuity correction. The *z*-test was used for the SES variable and was conducted as asymptotic linear-by-linear association test, to account for the ordering in the data. The *t* tests were used for continuous data and were conducted as Welsh’s two-sample *t* tests. Tests were conducted two-sided. SES was calculated based on the ACORN classification system (http://www.caci.co.uk)^61^. Prior psychiatric history was measured with the Schedule for Affective Disorders and Schizophrenia for School-Age Children (Present and Lifetime Version)^62^ and included learning disabilities, clinical sub-threshold diagnoses and deliberate self-harm at age 14; and clinical sub-threshold diagnoses and deliberate self-harm, but not learning disabilities, at age 17.

^a^Please note, the descriptive statistics are not based on the imputed data, which is why some participants have missing data on some descriptive variables, and accordingly some numbers do not add up.

#### Variable estimation

We computed the RFs based on unidimensional confirmatory factor analyses (CFAs; except for expressive suppression as this was assessed with only one item). We use factor scores and not sum scores to evade tau-equivalence and to decrease measurement error as much as possible (for a rationale and explanation see Additional file V Part A in Fritz et al.^18^). As all items ranged between three and six answer categories, we used categorical CFAs with a weighted least square mean and variance adjusted (WLSMV) estimator. The distress factor was similarly estimated using a longitudinal, unidimensional, categorical CFA (also with the WLSMV estimator), and was identified according to the strongly invariant model described by Wu and Estabrook (for a more detailed rationale see Supplementary I)^47^. We estimated all CFAs for the 1188 participants and pooled the results across the ten imputation sets. We then extracted the factor scores, that were pooled over the ten imputation analyses, and used those for the main analyses. For the main analyses we could include 1130 of the 1188 participants, as those had assessed information for CA. For completeness, we performed all analyses also on non-imputed data which can be found in Additional File I.

#### Prediction analyses

First, we performed a series of multiple linear regressions to predict distress at age 17. The first two models functioned as baseline models, one only included CA (model B1) and the other one included CA and gender as regressors (model B2). The next three models were the main models of interest: All contained CA and gender as regressor, the first model additionally contained the ten RFs (model M1), the second model additionally contained distress at age 14 (model M2), and the third model additionally contained both the RFs and distress at age 14 (model M3). Those analyses were performed to examine the directionality of the regressors (i.e. ± sign of the *b* values) and to investigate which regressors add significant variance to the explanation of distress at age 17. We additionally compared the models against each other using Likelihood-Ratio tests. Moreover, we re-estimated the models separately for the CA+ and the CA− groups as well as for males and females, to explore group effects.

Second, we aimed to disentangle the relative importance (RI) of the regressors in explaining general distress at age 17. Disentangling the RIs is of particular importance when the regressors are (or are assumed to be) strongly correlated, as every order of regressors then results in a different decomposition of sum of squares^48^. Here, we examined the RI metric “lmg” (cf. Lindeman, Merenda and Gold)^49^, which calculates sequential *R*^2^s while permuting and then averaging over the regressor orders^48^. To this end, we performed the three above described main models (M1, M2 and M3) as RI analyses. Moreover, we repeated the analyses separately for the CA+ and the CA− group as well as for males and females, to investigate differences in result patterns between subgroups.

Third, we conducted prediction analyses, to test with what accuracy the RFs and general distress at age 14 predict distress at age 17. We again used the three main models described above (M1, M2 and M3). All three prediction models were conducted once as a *categorical* model, with general distress at age 17 as categorical outcome variable, and once as *linear* models, with general distress at age 17 as a continuous outcome variable. For the categorical distress variable we conducted a series of factor mixture models^29^, which are hybrid models that add latent classes on top of the latent factors, with different invariance levels between the classes. We did this to classify the adolescents based on their distress profiles into categorical distress classes, while also taking into account the continuous nature of distress. Firstly, we applied latent class analyses to identify possible class solutions and then conducted one-factor mixture models with the appropriate class solutions (factor mixture model analyses details can be found in Supplementary II). For a factor mixture model solution with two classes we planned to use *logistic* prediction models, for a factor mixture model solution with three or more unordered classes we planned to use *multinomial* prediction models, and for a factor mixture model solution with three or more ordered classes we planned to use *ordinal* prediction models. For the prediction analyses the sample was quasi-randomly split into a training sample (75%; *n* = ~850) and a testing sample (25%; *n* = ~280; quasi-randomly means that that the relative class proportion of age-17 distress was kept equal between the training and the testing sample). We chose to have a larger training than testing sample, to be able to estimate as accurate prediction models as possible, particularly given that categorical prediction models require a substantial amount of power (relatively more than linear models, depending on the category number and size of the outcome variable). To determine the best link function for the categorical prediction models (i.e. logistic or probit) we used the Akaike information criterion and the residual deviance as model comparison indices. We then used the models resulting from the training procedures to predict distress at age 17 in the testing sample. To evaluate *categorical* prediction models, we calculated the amount of predicted distress scores that were predicted into their observed distress class. To evaluate the *linear* prediction models, we used the standard errors (SEs) of the age-17 distress factor scores and computed person-specific 95% confidence intervals (CI). We then calculated for how many adolescents our model could predict distress scores that fell into their respective 95% factor score CI. We again, also computed the analyses separately for the CA+ and the CA− group as well as for males and females, to investigate differences in result patterns between subgroups. This time, we could quantify the differences between the CA and the gender subgroups using proportion comparison tests, as we could describe the determined accuracies as accuracy proportions.

#### Software

Most analyses were performed in R version 3.5.1 (R packages are reported in Supplementary III)^50^. The factor scores and SEs for age-14 and age-17 distress were estimated in MPlus 8.2^51^, as it was not possible to compute the SEs based on categorical data in R. Similarly, we performed the latent class and factor mixture model analyses in MPlus as this allowed us to specify the items as categorical^51^.

## Results

### Sample

As none of the adolescents qualified as outlier in the multivariate space, we could include 1130 adolescents of which 631 were exposed (CA+) and 499 were not exposed to prior CA (CA−; see Table 1). The CA groups did not differ in age or gender proportions. SES was higher and a prior psychiatric history was less likely in the CA− than in the CA+ group. Of the 1130 participants, 620 were female and 510 male. The male and the female groups did neither differ in age nor SES. Female adolescents were more likely to have a prior psychiatric history.

### Disentangling the amount of variance that RFs and age-14 distress explain in age-17 distress

First we performed two baseline models, one only included CA (model B1) and the other one CA and gender (model B2) as predictors for age-17 distress. Then we conducted three main models. In addition to CA and gender, the first model contained the ten RFs (model M1), the second model contained age-14 distress (model M2), and the third model contained both the RFs and age-14 distress (model M3) as predictors for age-17 distress. We conducted the three main models for two reasons. Firstly, when comparing the individual effects of the RFs (M1) with the individual effects of age-14 distress (M2) it is possible to find out whether RFs and age-14 distress are similarly predictive for subsequent age-17 distress. This comparison seemed important, as the predictive value of previous distress on future distress has often been investigated, but little is known about the predictive magnitude of the RFs. Secondly, exploring the effects of RFs on age-17 distress over and above the effects of age-14 distress (M3) seemed relevant, as it gives an indication for the magnitude with which RFs explain change in distress between age 14 and 17.

Adding the RFs to CA and gender significantly improved the model and increased the explained variance from 4 to 20% (see Likelihood-Ratio test for M1 in Table 2). Similarly, adding age-14 distress (instead of the RFs) to CA and gender significantly improved the model and increased the explained variance from 4 to 23% (see Likelihood-Ratio test for M2 in Table 2; see Supplementary IV for Figures depicting change in distress). Adding age-14 distress to the model with CA, gender and the RFs improved the model significantly and increased the explained variance from 20 to 24% (see Likelihood-Ratio test for M3-D14 in Table 2). Adding the RFs to the model with CA, gender and age-14 distress increased the explained variance from 23 to 24%, but did not improve the model significantly (*p* = 0.07; see Likelihood-Ratio test for M3-RFs in Table 2). Hence, the RFs seemed to explain age-17 distress significantly, but seemed to explain the change in distress from age 14 to age 17 at best marginally. Importantly, there was no multicollinearity between the RFs and age-14 distress (see Supplementary V). When computing the analyses separately for the CA+ and the CA− group (CA+: M1 = 17%, M2 = 19%, M3 = 21%; CA−: M1 = 22%, M2 = 25%, M3 = 26%), or for females and males (females: M1 = 19%, M2 = 24%, M3 = 26%; males: M1 = 20%, M2 = 20%, M3 = 23%), the result patterns remained similar.

**Table 2:** Linear regression models.

	Model	*b*	*p* value	*R*^2^	*R*^2^ adj	LRT (df)	*p* value
Baseline model I (B1): with CA as regressor							
B1	CA	0.44	<0.001	3%	3%		
Baseline model II (B2): adding gender to B1							
						Compared against B1	
B2	Gender	−0.30	<0.001	4%	4%	25.33 (1)	<0.001
Adding RFs and age-14 distress (D14) separately to B2							
						Compared against B2	
M1	RFs	−0.86	–	20%	19%	297.25 (10)	<0.001
						Compared against B2	
M2	D14	0.63	<0.001	23%	23%	357.07 (1)	<0.001
Adding RFs and age-14 distress (D14) together to B2							
						Compared against M2	
M3	RFs	−0.19	–	24%	23%	22.11 (10)	0.07
						Compared against M1	
M3	D14	0.54	<0.001	24%	23%	81.93 (1)	<0.001

There is no *p* value for the RFs in model M1 and M3, as the bs of the RFs are here summed up to illustrate whether the cumulative effect is positive or negative, but as the RFs are ten individuals regressors there is no cumulative *p* value.

*adj* adjusted, *LRT* Likelihood-ratio test.

### Disentangling the relative importance of RFs and age-14 distress in explaining age-17 distress

We next decomposed the individual variance contribution of the regressors. In the model including both age-14 distress and the RFs, the RFs explained more variance in age-17 distress than age-14 distress (M3 RFs total variance = 57%; M3 age-14 distress total variance = 37%; see Table 3). Moreover, when taking age-14 distress into account the importance ranking of the RFs stayed the same as in the model without age-14 distress (i.e. compare M1 and M3). The self-esteem and brooding RFs explained most and expressive suppression explained the least amount of variance. The results pattern remained comparable when being computed separately for CA+ (M3 RFs total variance = 62%; M3 age-14 distress total variance = 37%) and CA− groups (M3 RFs total variance = 58%; M3 age-14 distress total variance = 39%), as well as for female (M3 RFs total variance = 54%; M3 age-14 distress total variance = 43%) and male participants (M3 RFs total variance = 63%; M3 age-14 distress total variance = 30%).

**Table 3:** Relative importance analyses for the whole group: for RFs only (M1), age-14 distress only (M2), and RFs and age-14 distress together (M3).

Variable	% M1: RFs only	Bootstrap CI	% M2: D14 only	Bootstrap CI	% M3: RFs and D14	Bootstrap CI
Abs	19.94	–	23.10	–	24.27	–
CA	06.74	02.03–13.11	08.37	03.34–15.52	05.00	01.47–10.05
Gender	02.30	00.58–06.28	03.16	01.18–07.28	01.37	00.49–04.08
Total	09.03	–	11.53	–	06.37	–
Neg. self-esteem	21.12	14.84–28.31	–	–	12.58	09.80–16.18
Pos. self-esteem	17.79	10.64–24.67	–	–	11.25	06.64–16.28
Brooding	16.10	09.86–23.02	–	–	09.58	06.56–13.80
Family cohesion	08.72	04.42–14.17	–	–	05.71	02.88–09.55
Aggression	07.03	02.30–13.95	–	–	04.11	01.54–08.93
Friendships	05.81	02.47–10.77	–	–	03.60	02.06–06.69
Family support	05.03	02.17–10.13	–	–	03.44	01.50–07.08
Reflection	04.50	02.55–08.10	–	–	03.05	02.07–05.15
Dis. tolerance	04.17	01.31–08.45	–	–	02.79	00.98–05.85
Expressive sup.	00.72	00.10–03.62	–	–	00.43	00.06–02.58
Total	90.97	–	–	–	56.55	–
D14	–	–	88.48	80.44–94.15	37.08	28.76–43.14
Total	–	–	88.48	–	37.08	–

*D14* age-14 distress, *CI* confidence interval, *Abs* absolute amount of explained variance, *CA* childhood adversity, *Neg.* negative, *Pos.* positive, *Dis.* distress, *sup.* suppression.

### Disentangling the accuracy with which RFs and age-14 distress predict age-17 distress

**Fig. 1:**
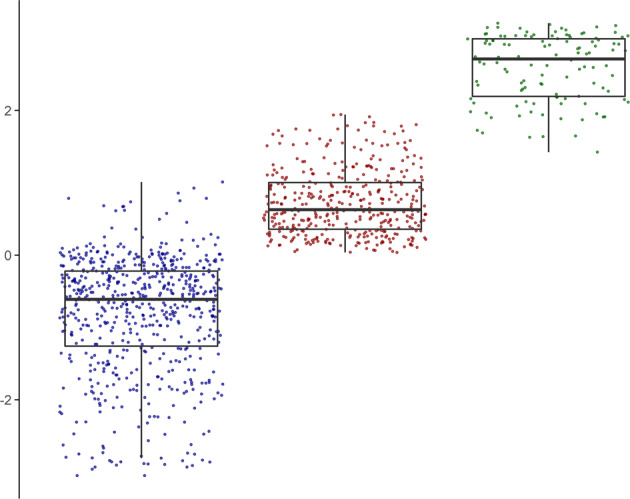
Three-class factor mixture model distress solution (*x*-axis) plotted against the continuous distress severity scores (*y*-axis). Left boxplot = low/mild distress class (*n*  =  623); middle boxplot = moderate distress class (*n*  =  390); right boxplot = high distress class (*n*  =  117). Centre line = median (50% quantile); lower box limit = 25% quantile; upper box limit = 75% quantile; lower whisker = smallest observation greater than or equal to the lower box limit − 1.5 × inter quartile range (IQR); upper whisker = largest observation less than or equal to the upper box limit + 1.5 × inter quartile range (IQR).

We first performed a series of factor mixture models to classify the adolescents based on their categorical distress profiles, while also taking into account the continuous nature of distress. The three-class model, which allows the factor score mean to vary per distress class (called factor mixture model-1; for more specific analysis details see Supplementary II), performed well (entropy = 0.97) and revealed a theoretically plausible solution, splitting the adolescents into “low/mild”, “moderate” and “high” distress severity classes. Figure 1 shows the class solution plotted against the continuous general distress scores.

As the best class solution was ordered categorical, we conducted three *ordinal* prediction models with the three-class distress variable as outcome variable. Of the three models one again contained the RFs (M1), one age-14 distress (M2), and one both (RFs and age-14 distress; M3) in addition to gender and CA as predictors. Here, we conducted the three models to investigate whether RFs (M1) have a similar predictive accuracy as age-14 distress (M2), and to find out whether the combination of RFs and age-14 distress is better than one information source alone (M3 vs. M1 and M2). The applied ordinal regression models have a proportional odds assumption, which was not met for all predictors. Therefore, we conducted the ordinal regressions as partial proportional odds models and relaxed the proportional odds assumption for those predictors that did not meet the assumption (see details in Supplementary VI).

The three models (M1–M3) had a low to acceptable accuracy ranging from 62 to 64% (see Table 4). Hence, about 2 out of 3 adolescents were correctly predicted into their distress severity class, regardless of using RFs, age-14 distress, or both as predictors for age-17 distress. Once more, the results were generally comparable when we split the adolescents into CA+ (accuracy: M1 = 54%, M2 = 60%, M3 = 58%), CA− (accuracy: M1 = 66%, M2 = 69%, M3 = 69%), female (accuracy: M1 = 58%, M2 = 59%, M3 = 58%) and male groups (accuracy: M1 = 61%, M2 = 64%, M3 = 61%). More specifically, the prediction accuracy did not differ between the CA and gender subgroups (for details see Supplementary VII); only model M1 revealed a borderline effect for the CA+ vs. CA− comparison (Chi^2^ = 3.82, df = 1, *p* = 0.051).

**Table 4:** Ordinal prediction analyses for the whole group: for RFs only (M1), age-14 distress only (M2), and RFs and age-14 distress together (M3).

	M1: RFs only		M2: D14 only		M3: RFs and D14	
	Observed	Predicted	Observed	Predicted	Observed	Predicted
Residual deviance	1420.35	–	1390.41	–	1392.83	–
ROC	–	Low = 0.70 Mod = 0.65 High = 0.75	–	Low = 0.69 Mod = 0.68 High = 0.71	–	Low = 0.69 Mod = 0.68 High = 0.74
Sensitivity	–	Low = 0.83 Mod = 0.54 High = 0.00	–	Low = 0.79 Mod = 0.53 High = 0.00	–	Low = 0.82 Mod = 0.53 High = 0.00
Specificity	–	Low = 0.52 Mod = 0.79 High = 1.00	–	Low = 0.50 Mod = 0.76 High = 1.00	–	Low = 0.52 Mod = 0.77 High = 1.00
Accuracy	–	0.64 Low = 0.68 Mod = 0.66 High = 0.50	–	0.62 Low = 0.65 Mod = 0.64 High = 0.50	–	0.63 Low = 0.67 Mod = 0.65 High = 0.50
Low distress severity	155	189 of which —129 correct —44 false mod —16 false high	155	186 of which —123 correct —46 false mod —17 false high	155	187 of which —127 correct —45 false mod —15 false high
Mod distress severity	97	91 of which —52 correct —26 false low —13 false high	97	95 of which —51 correct —32 false low —12 false high	97	93 of which —51 correct —28 false low —14 false high
High distress severity	29	1 of which —00 correct —00 false low —01 false mod	29	0 of which —00 correct —00 false low —00 false mod	29	1 of which — 00 correct — 00 false low — 01 false mod

All models were computed with childhood adversity and gender as predictors. Variable for which the proportional odds assumption was relaxed can be found in Supplementary VI. *D14* age-14 distress, *Mod* moderate, *ROC* receiver operating characteristic, *Accuracy* relative number of correctly predicted cases, *Sensitivity* e.g. for low distress: the number of adolescents who are correctly predicted into the low distress group divided by all adolescent who are actually in the low distress group, *Specificity* e.g. for low distress: the number of adolescents who are correctly not predicted into the low distress group divided by all adolescent who are actually not in the low distress group.

We next tested the prediction accuracy for *linear* models with the continuous distress severity variable as outcome measure. These analyses revealed that in contrast to the *ordinal* models, the prediction accuracy for all three *linear* models was low (37 to 41%; Table 5), as the age-17 distress level of only about two in five adolescents was predicted accurately. Similar findings were revealed when splitting the group based on CA (CA+: M1 = 34.62%, M2 = 32.69%, M3 = 36.54%; CA−: M1 = 40.32%, M2 = 38.71%, M3 = 38.71%) and gender (female: M1 = 32.90%, M2 = 35.53%, M3 = 34.87%; male: M1 = 38.89%, M2 = 41.27%, M3 = 42.06%). Once more, the prediction accuracy did not differ significantly between the CA and gender subgroups (see Supplementary VII).

**Table 5:** Linear prediction analyses for the whole group: for RFs only (M1), age-14 distress only (M2), and RFs and age-14 distress together (M3).

	M1: RFs only		M2: D14 only		M3: RFs and D14	
	Observed	Predicted	Observed	Predicted	Observed	Predicted
RMSE	1.18	1.18	1.15	1.13	1.16	1.13
*R*^2^	0.18	0.16	0.23	0.22	0.22	0.22
MAE	0.90	0.91	0.87	0.88	0.88	0.88
Accuracy	–	37.14%	–	40.00%	–	40.71%
Predicted into CI_95%_	–	104	–	112	–	114
Not predicted into CI_95%_	–	176	–	168	–	166
Accuracy plots *x*-axis: Observed (left = black) vs. predicted (right = grey) distribution *y*-axis: Distress level						

All models were computed with childhood adversity and gender as predictors. Model accuracy was based on 1000 bootstraps. *D14* age-14 distress, *RMSE* root mean squared error, *MAE* mean absolute error, *Accuracy* relative number of correctly predicted cases.

### Post-hoc exploration: disentangling the accuracy for fewer RFs predicting age-17 distress

In our RF regression models (i.e. the M1s), three RFs were (at least marginally (<0.10)) significant in three of the four subgroups, namely negative self-esteem, positive-self-esteem, and brooding. Moreover those three RFs had in three of the four subgroups the highest RI. Therefore, we next re-ran all prediction models this time instead of including all ten RFs, CA and gender, only including these three RFs and gender. We did this to investigate whether the assessment of just three RFs and gender would provide similar information as all ten RFs, CA and gender (i.e. M1). This is important, as such an assessment may be more feasible and efficient in many non-clinical settings (e.g. in school assessments). Interestingly, in these post-hoc analyses, both the ordinal and the linear models performed similar as the models including all RFs (change in accuracy: ordinal models from 64 to 62%, Chi^2^ = 0.37, df = 1, *p* = 0.54; linear models from 37.14 to 37.14%, Chi^2^ = 0, df = 1, *p* = 1). Moreover, the models including gender, the three RFs and age-14 distress were rather comparable to the models including gender, CA, all 10 RFs, and age-14 distress (i.e. M3; change in accuracy: ordinal models from 63 to 60%, Chi^2^ = 0.61, df = 1, *p* = 0.44; linear models from 40.71 to 40.71%, Chi^2^ = 0, df = 1, *p* = 1). For completeness, we also conducted the prediction analyses with a subset of the RFs separately in the subgroups, which can be found in Supplementary VIII.

## Discussion

We aimed to shed light onto potentially promising RF targets that reduce subsequent distress, by pursuing three sub-goals: *First*, we intended to find out to which degree RFs can explain subsequent distress. Our results suggest that RFs explained a similar amount of variance in age-17 distress as age-14 distress could explain, and when the predictors were used together RFs even explained a higher amount of variance than age-14 distress. *Second*, we aimed to find out which RFs are the best indicators for subsequent distress. Our results showed that self-esteem and brooding RFs explained most variance and revealed significance in the multivariable regression models. Aggression explained less variance but was still a significant predictor. *Third*, we intended to explore with what accuracy RFs can predict distress levels 3 years later. We found that RFs and distress at age 14 were similarly accurate in predicting distress at age 17. The prediction accuracy was low and highly unsatisfactory when we predicted continuous distress scores. When we predicted more crude ordinal (“low”, “moderate” and “high”) distress classes the accuracy was again not good, but acceptable. As such, both RFs and distress at age 14 (as well as their combination) are able to correctly predict the categorical distress class of about 2 in 3 adolescents.

RFs and/or age-14 distress explained about one-fifth to one-fourth of the overall variance in distress 3 years later. Importantly, this was after CA and gender were taken into account. Hence, despite the fact that we have used gender, life-history information (i.e. CA), a broad range of distress symptoms and as many as ten empirically-supported RFs, we were only able to explain a small proportion of the variance in distress 3 years later. This is alarming and interesting at the same time. Dinga et al.^7^ put forward the explanation that the way psychopathology is defined may lack important information (i.e. content validity), such as biological components, which may make it so difficult to predict it well. Another explanation could be derived from the time period we have investigated. We assessed the adolescents during early (age 14) and later (age 17) adolescence, which is generally described as a particularly malleable period during which a lot of mental health problems develop^3^. That is, distress predictions over a period during which many mental health problems manifest themselves may be particularly difficult. A third account may come from the instructions that were provided for the assessment of the distress symptoms: “please tick how often you have felt or acted in this way over the past 2 weeks”. The instructions assess distress during the past 2 weeks, which for some adolescents may have captured state- rather than trait-distress. An outcome construct that at least to some extent captures state characteristics may complicate the prediction even further. In sum, insufficient content validity, a sensitive developmental time period, and state-like characteristics of the distress variable may all help explain why it was so difficult to predict subsequent distress.

While the RFs explained age-17 distress significantly, the RFs explained change in distress from age 14 to age 17 at best marginally. Yet, the importance ranking of the RFs for explaining age-17 distress did not change when taking age-14 distress into account. Importantly, there was no overlap between RFs and distress items content-wise, and no multicollinearity between RFs and age-14 distress. RFs not only had a higher RI than age-14 distress when both predictors were used to explain age-17 distress, but the RFs and age-14 distress had a similar accuracy for predicting age-17 distress. This clearly is a notable finding, as RFs could similarly well predict distress over the course of 3 years, as distress could predict itself over the course of 3 years. Moreover, a combination of the two information sources (RFs and age-14 distress) did not necessarily seem advantageous above either source alone. Therefore, if our results were to be replicated, we would assume that knowledge on the RFs may, due to its “conceptual commitment to strengths and assets” (see^52^, p. 136), be highly interesting for various public health and clinical settings. More specifically, in settings where a strengths-focus would be more feasible than a symptom-focus, RFs could be assessed to screen, monitor and potentially promote mental health.

If we would have to judge which of the RFs may be the most promising for screening, monitoring and potentially promoting mental health, we probably would choose brooding and the self-esteem RFs, as those three had the strongest RI in reducing the risk of subsequent distress and were significant in the multivariable RF models (M1). Importantly, our prediction results remained rather stable when we used only those three instead of all ten RFs. Moreover, those three RFs together are measured with only 15 items. Hence, assessing brooding and self-esteem RFs would not only have a relatively low stigma risk, but would also be highly time and money efficient. The finding that both self-esteem and brooding seem to play such an important role in the development of mental health problems has been noted in previous research and has led to the suggestion to use self-esteem^53^ or brooding^54^ as time-efficient and less stigma-prone mental health screens. Young and Dietrich^54^ for example employed the same brooding subscale as used in our study (five items of the RRS)^38^ and detected a screening accuracy of 91 percent for concurrent depressive symptoms in young adolescents. Moreover, both self-esteem and brooding have already been found to be successful intervention targets^55,56^, particularly for interventions aimed at reducing internalising disorders and/or increasing mental well-being. Interventions targeting self-esteem are suggested to be most successful when provided earlier during adolescence, as self-esteem often is more amenable during early than during late adolescence^53^. Moreover, rumination focused cognitive behaviour therapy has been shown to be a promising prevention intervention for adolescents at risk for internalising mental health problems^56^. Yet, our results require replication in an independent sample and need ideally to be tested in translational studies, before screening and intervention-related recommendations can be made. Moreover, additional replication in other populations would be ideal, to ensure a clear scope for generalisation.

It is important to note that our *linear* prediction models, which are derived from the group level, are not good enough to predict individual-level distress scores 3 years later. Those models translated for only two in five adolescents correctly to the individual level. Our *categorical* prediction models, which are also derived from the group level, did predict individual-level distress severity classes better, but there is still plenty of room for improvement. Those models translated for about two-third of the adolescents correctly to the individual level. Hence, the generalisation from group to individual level is limited, particularly when predicting *continuous* transdiagnostic distress severity. Therefore, it is crucial that future research identifies ways to increase the prediction accuracy for subsequent distress severity. In sum, we recommend that future research (a) examines whether our findings replicate, (b) tests additional RFs that were not measured in our adolescent cohort but are empirically found to reduce subsequent distress, (c) identifies ways which further increase the prediction accuracy (e.g. shorter prediction intervals), (d) is conducted at the individual rather than (or in addition to) the group level, and (e) explores in which prevention and intervention settings targeting RFs may be most helpful.

Last but not least, our study is not without limitations. First, ROOTS has a slightly higher than average SES and thus may mainly generalise to more wealthy populations^30^. Second, our latent class and factor mixture model analyses were based on a grandmedian imputation data set, rather than being conducted separately on the ten imputation data sets, as there is no method and consensus yet on how to pool over class solutions (for details see Supplementary II). Third, the binary CA variable may not be ideal as it omits the type of the adversity experience, as well as its severity and frequency. Particularly CA severity may be a valuable consideration and addition in future research^57^. However, justification for using CA as a binary indicator stems from research showing that CAs are likely to co-occur and that clustered CA indices have a robust, negative effect on mental health problems^32,46,57^. For future research it would be ideal if adversity would also be assessed, and controlled, for the interim period between the assessment of the RFs and the assessment of subsequent mental distress. Fourth, the RFs were not all assessed with measures developed to particularly reflect the RF construct at hand (e.g. aggression or expressive suppression). Hence, future research should aim to replicate our results with scales particularly developed for the specific RFs, to increase the content validity. Fifth, we only tested ten RFs, as only those were assessed in our adolescent cohort. However, in the realm of complexity we think that it would be advantageous if future research could assess and test more than ten empirically-supported RFs. Sixth, our distress index was mainly defined by internalising (and not externalising) symptoms and does not contain information on the distress chronicity. Seventh, we built the prediction models on a subset of the ROOTS cohort (n ~850) to predict distress 3 years later for another ROOTS subset (*n* ~280). This means that we used data from the same cohort for training and testing our model. However, it may be that adolescents in our cohort are more comparable to each other than to the general population. This would mean that our prediction accuracy would be lower when using our model to predict distress scores for adolescents who did not take part in ROOTS. Therefore, replication of our findings in a different sample is crucial. Eights, here we mainly focussed on the overall sample and not so much on findings within the subgroups (CA+ vs. CA−, females vs. males). Yet, there were slight differences in the RI of the RFs between the subgroups. Future research should more specifically focus on those differences, for example with moderation analyses.

Critics might argue that investigating age-17 distress as both a categorical and a continuous outcome is superfluous. Yet, we believe that there are good reasons from a scientific as well as a clinical point of view that justify the usage of both (categorical and continuous outcomes) in conjunction. From a statistical point of view it may perhaps seem neater to investigate distress continua. But, first of all our distress classes did take the distress continuum into account, and more importantly, as prior research often only looked at categorical outcomes we feel that it is high time to gain information on the comparison of precise continuous versus more crude categorical outcomes. As our findings showed, it seems like we are not good enough yet to predict precise distress continua, but we are getting into an acceptable range for predicting crude distress classes (from either RFs, distress, or their combination). From a translational point of view, one may favour a categorical outcome as this is often used in clinics, such as cut-offs like “low risk”, “at risk/sub-threshold”, and “diagnosed”. Although crude categorical outcomes may be more easily translatable, providing results of both approaches has given rise to the clinically relevant finding that RFs and prior distress may be promising targets for screens aiming at predicting rough distress risk-categories (e.g. “low”, “moderate”, “high”), but not yet for screens aiming at predicting precise distress risk levels.

As pointed out in the introduction, there is a sparse but ongoing discourse about whether resilience and risk factors are opposing sides of the same coin, which cannot fully be done justice within the scope of this manuscript. However, we suggest that future studies could conduct more idiographic rather than group level research, as the “relationship between resilience and risk factors is likely to additionally depend on biological predispositions, type(s) of adversity experienced, the specific environmental circumstances, and the developmental stage” (see p. 3 in Additional file XVI of Fritz et al.^18^) of the adolescent. Moreover, while this manuscript specifically focusses on using RFs that predict mental health problems (in individuals with and without CA exposure), it would be interesting to see future research taking the same modelling approach but focussing on those factors that predict a resilient functioning outcome. To this end one could for example focus on resilience predictors reviewed by Kalisch et al. (including hair cortisol concentration, trait self-enhancement, expression of specific gene networks, and cortisol stress reactivity)^58^, on factors that predict resilient growth trajectories and resilient functioning outcomes as reviewed by Bonanno et al. (including perceived control, high positive affectivity, low negative affectivity, trait resilience, low brooding, coping self-efficacy, emotional support, social support, instrumental support, favourable worldviews, and positive emotions)^59^, or on factors that relate to resilient functioning specifically following childhood maltreatment, as reviewed in Ioannidis et al. (including the social environment as well as biological factors related to the hypothalamic–pituitary–adrenal axis and polygenetics)^60^.

Overall, our results showed that the RFs were able to correctly predict the categorical (‘low’/‘moderate’/‘high’) distress class of 2 in 3 adolescents 3 years later. This finding was highly similar when predicting age-17 from age-14 distress. The three RFs that were most promising in predicting and reducing subsequent distress were positive self-esteem, negative self-esteem and brooding. Hence, those three RFs may potentially be promising targets for risk-detection and interventions, if they hold up in replication and translational research.

## Data Availability

Data for this specific paper has been uploaded to the Cambridge Data Repository 10.17863/CAM.46642 and is password protected. Our participants did not give informed consent for their measures to be made publicly available, and it is possible that they could be identified from this data set. Access to the data supporting the analyses presented in this paper will be made available to researchers with a reasonable request to openNSPN@medschl.cam.ac.uk.

## Supplementry information

Supplementry information Supplementry information2

## Code availability

Analysis code is available from http://jessica-fritz.com/.
