# Supplementary material for: Embracing the positive: an examination of how well resilience factors at age 14 can predict distress at age 17
Source: Transl Psychiatry. 2020 Aug 5;10:272. doi: 10.1038/s41398-020-00944-w (PMC7406495; doi:10.1038/s41398-020-00944-w)
Supplement: Supplementary file 1 — Supplementry information [file 41398_2020_944_MOESM1_ESM.pdf]

Supplements for the Manuscript

"Embracing the positive: An examination of how well resilience factors at age 14 can predict distress at age 17"

J. Fritz,[1,*] J. Stochl,[1,2] I.M. Goodyer,[1] A.-L. van Harmelen,[1,A] & P.O. Wilkinson[1,A]

* Correspondence: Jessica Fritz, jf585@cam.ac.uk
[1] University of Cambridge, Department of Psychiatry, United Kingdom
[2] Charles University, Department of Kinanthropology, Czech Republic
[A] shared last author

Supplement I

For the confirmatory factor analyses (CFAs) we pooled the factor scores over the 10 result sets (i.e. one for each imputation data set). We used modification indices only when statistically necessary and theoretically defensible. All CFA models fitted reasonably. For aggression the resulting factor scores were notably poorly distributed and we therefore binarized this variable. The continuous latent distress scores used in the manuscript are based on a strongly invariant, categorical CFAs (i.e. L+T+I IM models in the below table), to ensure the latent mean comparability between distress at age 14 and age 17. More specifically, we applied the delta parametrization, equated item loadings and item thresholds across the two time points (i.e. age 14 and 17), fixed all item intercepts to 0, the item scales of the first time point to 1, the latent factor mean of the first time point to 0, and the latent factor variance of the first time point to 1.

(Longitudinal) Confirmatory Factor Analyses Conducted with WLSMV estimator, n = 1188

Model	Robust CFI	Robust TLI	Robust RMSEA	RMSEA 90% CI	SRMR	Chisq(df)
Friendship support* [1], *5 items, 1 unique item covariance						
**BM**	0.988	0.969	0.067	0.043– 0.093	0.036	12.652(4)
Family support* [2], *5 items, 1 unique item covariance						
**BM**	0.995	0.987	0.062	0.039-0.088	0.023	9.285(4)
Family cohesion* [2], *7 items, 1 unique item covariance						
**BM**	0.980	0.967	0.070	0.057-0.085	0.042	48.773(13)
Positive self-esteem* [3], *5 items, 1 unique item covariance						
**BM**	0.996	0.989	0.076	0.052-0.102	0.016	9.446(4)
Negative self-esteem* [3], *5 items, 0 unique item covariances						
**BM**	0.993	0.987	0.045	0.022-0.069	0.017	6.542(5)
Brooding* [4], *5 items, 0 unique item covariances						
**BM**	0.991	0.983	0.068	0.047-0.091	0.029	14.520(5)
Reflection* [4], *5 items, 1 unique item covariance						
**BM**	0.999	0.998	0.023	0.000-0.054	0.018	3.435(4)
Distress tolerance* [5], *5 items, 1 unique item covariance						
**BM**	0.977	0.942	0.128	0.105-0.153	0.051	36.272(4)
Aggression* [6], *4 items, 0 unique item covariances						
**BM**	0.988	0.965	0.029	0.000-0.071	0.036	1.387(2)
Distress* [7,8], *41 items, 2 unique item covariances						
**C IM 1**	0.988	0.987	0.026	0.025-0.027	0.043	
**C IM 2**	0.988	0.988	0.026	0.025-0.027	0.043	
**C IM 3**	0.989	0.989	0.026	0.025-0.027	0.043	
**C IM 4**	0.989	0.989	0.026	0.025-0.027	0.043	
**C IM 5**	0.988	0.987	0.027	0.026-0.028	0.045	
**C IM 6**	0.989	0.988	0.026	0.025-0.027	0.044	
**C IM 7**	0.990	0.990	0.026	0.025-0.027	0.044	
**C IM 8**	0.986	0.986	0.027	0.026-0.028	0.047	
**C IM 9**	0.989	0.988	0.026	0.025-0.028	0.045	
**C IM 10**	0.988	0.988	0.026	0.025-0.027	0.045	
**L+T+I IM 1**	0.986	0.986	0.027	0.026-0.028	0.042	
**L+T+I IM 2**	0.987	0.987	0.027	0.026-0.028	0.042	
**L+T+I IM 3**	0.988	0.988	0.027	0.026-0.028	0.042	
**L+T+I IM 4**	0.988	0.988	0.027	0.026-0.028	0.043	
**L+T+I IM 5**	0.986	0.986	0.028	0.027-0.029	0.045	
**L+T+I IM 6**	0.987	0.987	0.027	0.026-0.028	0.043	
**L+T+I IM 7**	0.989	0.989	0.027	0.026-0.028	0.043	
**L+T+I IM 8**	0.985	0.984	0.028	0.027-0.029	0.046	
**L+T+I IM 9**	0.988	0.988	0.027	0.026-0.028	0.044	
**L+T+I IM 10**	0.987	0.987	0.027	0.026-0.029	0.044	

Note. WLSMV = weighted least squares mean and variance corrected estimator; CFI = Comparative fit index; TLI = Tucker-Lewis index; RMSEA = Root mean square error of approximation; CI = Confidence interval; chisq = chi-square; BM = baseline model; C IM = configural invariance model; L+T+I IM = loadings, thresholds, and intercepts invariance model. All models were conducted with the delta parameterization.

Supplement II

For the categorical prediction model we aimed to classify the adolescents based on their distress profiles into a categorical distress variable. Firstly, we applied latent class analysis (LCA) with ordinal items, an MLR estimator, and a logit link (see Table 1), to identify possible class solutions. We used the same 41 anxiety and depression items for the LCA as for the general distress factor model. The 2-class solution was significantly better than a 1-class model (Likelihood-Ratio test (LRT) = 22924.59, p < .001). We also tested 3- and 4-class solutions but those did not fit significantly better. Based on those results we conducted a series of factor mixture models (FMMs),[9] which are hybrid models that add latent classes on top of the latent factors, with different invariance levels between the classes. We tested those FMMs with 2, 3, and 4 classes. The FMM1 is the factor mixture model with the most invariance constraints between classes, as it only allows the factor mean to vary between classes. The FMM1 with 2 classes did not fit better than the FMM1 with 1 class (LRT = 19632.75, p = .746). The FMM1 with 3 classes fitted better than the FMM1 with 2 classes (LRT = 7608.02, p < .001). Similarly, the FMM1 with 4 classes fitted better than the FMM1 with 3 classes (LRT = 2157.44, p < .001), but had a lower entropy (0.970 vs 0.953). For the analyses with the non-imputed data, one class of the FMM1 4-class model only contained 42 adolescents, with only 10 being sampled in the test sample. This is already a small group to be predicted, but when we then split the sample further into CA+ vs CA- and into female vs male, the high distress class had for the CA- group only 6 adolescents in the training and 2 in the test sample. Similarly, the female group had only 5 adolescents in the training and 1 in the test sample. We therefore considered this class practically too small. We also tested the FMM2 model, in which in addition to the factor mean also the factor variance can vary between classes. The FMM2 solution with 2 classes fitted well. Yet, the FMM2 model could not successfully be fitted on the non-imputed data. We therefore decided not to go forward with the FMM2 models. In sum, we decided to go forward with the FMM1 3-class solution, to keep comparisons with and without imputed data possible and to have sufficiently predictable class sizes. Moreover, the FMM1 with 3 classes revealed a theoretically plausible and practical solution, which is described in the main text. For completeness we also computed the prediction analyses with the FMM1 with 4 classes as outcome variable, which can be found in Supplement IX.

Importantly, the categorical class solutions are not necessarily ordered categorical, but can be nominal. Consequently, it is not possible to pool over the class solutions of the 10 imputed data sets, as this would not take into account non-ordered class allocations. The FMM1 naturally results in an ordered categorical class solutions, with class-varying factor means. Yet, for the FMM2, for which we allow in addition to the factor mean also the factor variance to vary per class, the solution can be nominal. Similarly, LCA class results can also be content specific, and thus nominal, rather than ordered categorical. Therefore, we computed a grandmedian dataset, for which we took for each score the median value across the 10 imputed datasets. Based on this data set we then performed the LCA and FMM models. Using a grandmedian dataset is disadvantageous, as it does not take into account the between-imputation variance, yet, it preserves the interpretation of the classes, which was of particular interest here.

Latent Class Analyses with MLR estimator and logit link

classes	AIC	BIC	BIC_{adj}	Entropy	LMR LRT	p-value	Class counts
2	76466.74	77721.51	76936.95	0.996	22924.59	<.001	1=1006; 2=182
3	67746.95	69631.64	68453.21	0.973	08957.59	.765	1=647; 2=406; 3=135
4	65288.68	67803.29	66230.99	0.968	02703.20	.767	1=159; 2=504; 3=401, 4=124

Note. AIC =Akaike information criterion. BIC =Bayesian information criterion. BIC_{adj} = sample size adjusted BIC. LMR LRT = Lo-Mendel-Rubin adjusted likelihood ratio test for class comparisons.

One-Factor Mixture Models with MLR estimator and logit link

classes	AIC	BIC	BIC_{adj}	Entropy	LMR LRT	p-value	Class counts
FMM1: loadings = class invariant; thresholds = class invariant; factor mean = varying per class (fixed to 0 in 1 class for identification); factor variance = fixed to 0							
2	76847.72	77685.93	77161.82	0.998	19632.75	0.746	1=1018; 2=170
3	68169.12	69017.49	68487.03	0.970	07608.02	<0.001	1=412; 2=644; 3=132
4	65710.97	66569.49	66032.68	0.953	02157.44	<0.001	1=403; 2=480; 3=125; 4=180
FMM2: loadings = class invariant; thresholds = class invariant; factor mean = varying per class (fixed to 0 in 1 class for identification); factor variance = varying per class							
2	63744.30	64592.67	64062.21	0.989	349.03	<0.001	1=1072; 2=116
3[NI]	-	-	-	0.977	-	-	1=1040; 2=32; 3=116
4	63734.47	64613.32	64063.80	0.585	012.47	0.578	1=657; 2=381; 3=102; 4=48

Note. AIC =Akaike information criterion. BIC =Bayesian information criterion. BIC_{adj} = sample size adjusted BIC. LMR LRT = Lo-Mendel-Rubin adjusted likelihood ratio test for class comparisons. [NI] = the model was not identified.

Supplement III

Package (version number)	Reference
beanplot (1.2)	Peter Kampstra (2008). Beanplot: A Boxplot Alternative for Visual Comparison of Distributions. Journal of Statistical Software, Code Snippets 28(1). 1-9. http://www.jstatsoft.org/v28/c01/.[10]
brant (0.2-0)	Benjamin Schlegel and Marco Steenbergen (2018). brant: Test for Parallel Regression Assumption. R package version 0.2-0. https://CRAN.R-project.org/package=brant[11]
car (3.0-2)	John Fox and Sanford Weisberg (2011). An {R} Companion to Applied Regression, Second Edition. Thousand Oaks CA: Sage. http://socserv.socsci.mcmaster.ca/jfox/Books/Companion[12]
caret (6.0-81)	Max Kuhn (2018). Caret: Classification and Regression Training. https://CRAN.R-project.org/package=caret[13]
coin (1.2-2)	Torsten Hothorn, Kurt Hornik, Mark A. van de Wiel, Achim Zeileis (2008). Implementing a Class of Permutation Tests: The coin Package. Journal of Statistical Software 28(8), 1-23. http://www.jstatsoft.org/v28/i08/.[14]
dplyr (0.7.7)	Hadley Wickham, Romain François, Lionel Henry and Kirill Müller (2018). Dplyr: A Grammar of Data Manipulation. https://CRAN.R-project.org/package=dplyr[15]
foreign (0.8-70)	R Core Team (2017). Foreign: Read Data Stored by ‘Minitab’, ‘S’, ‘SAS’, ‘SPSS’, ‘Stata’, ‘Systat’, ‘Weka’, ‘dBase’, ... R package version 0.8-70. https://CRAN.R-project.org/package=foreign[16]
Hmisc (4.1-1)	Frank E Harrell Jr, with contributions from Charles Dupont and many others. (2018). Hmisc: Harrell Miscellaneous. https://CRAN.R-project.org/package=Hmisc[17]
MASS (7.3-50)	Venables, W. N. & Ripley, B. D. (2002) Modern Applied Statistics with S. Fourth Edition. Springer, New York. ISBN 0-387-95457-0[18]
mice (3.5.0)	Stef van Buuren, Karin Groothuis-Oudshoorn (2011). Mice: Multivariate Imputation by Chained Equations in R.

	Journal of Statistical Software, 45(3), 1-67. https://www.jstatsoft.org/v45/i03/.[19]
MLmetric (1.1.1)	Yachen Yan (2016). MLmetrics: Machine Learning Evaluation Metrics. https://CRAN.R-project.org/package=MLmetrics[20]
pastecs (1.3.21)	Philippe Grosjean and Frederic Ibanez (2018). Pastecs: Package for Analysis of Space-Time Ecological Series. https://CRAN.R-project.org/package=pastecs[21]
pROC (1.14.0)	Xavier Robin, Natacha Turck, Alexandre Hainard, Natalia Tiberti, Frédérique Lisacek, Jean-Charles Sanchez and Markus Müller (2011). pROC: an open-source package for R and S+ to analyze and compare ROC curves. BMC Bioinformatics, 12, p. 77. Doi:10.1186/1471-2105-12-77[22]
qgraph (1.5)	Sacha Epskamp, Angelique O. J. Cramer, Lourens J. Waldorp, Verena D. Schmittmann, Denny Borsboom (2012). Qgraph: Network Visualizations of Relationships in Psychometric Data. Journal of Statistical Software, 48(4), 1-18. http://www.jstatsoft.org/v48/i04/.[23]
relaimpo (2.2-3)	Ulrike Groemping (2006). Relative Importance for Linear Regression in R: The Package relaimpo. Journal of Statistical Software, 17(1), 1-27.[24]
reshape (0.8.8)	H. Wickham. Reshaping data with the reshape package. Journal of Statistical Software, 21(12), 2007.[25]
semTools (0.5-1.933)	Jorgensen, T. D., Pornprasertmanit, S., Schoemann, A. M., & Rosseel, Y. (2019). semTools: Useful tools for structural equation modelling. R package version 0.5-1.933. Retrieved from https://CRAN.R-project.org/package=semTools[26]
VGAM (1.1-1)	Thomas W. Yee (2010). The VGAM Package for Categorical Data Analysis. Journal of Statistical Software, 32(10), 1-34. http://www.jstatsoft.org/v32/i10/.[27]

Supplement IV

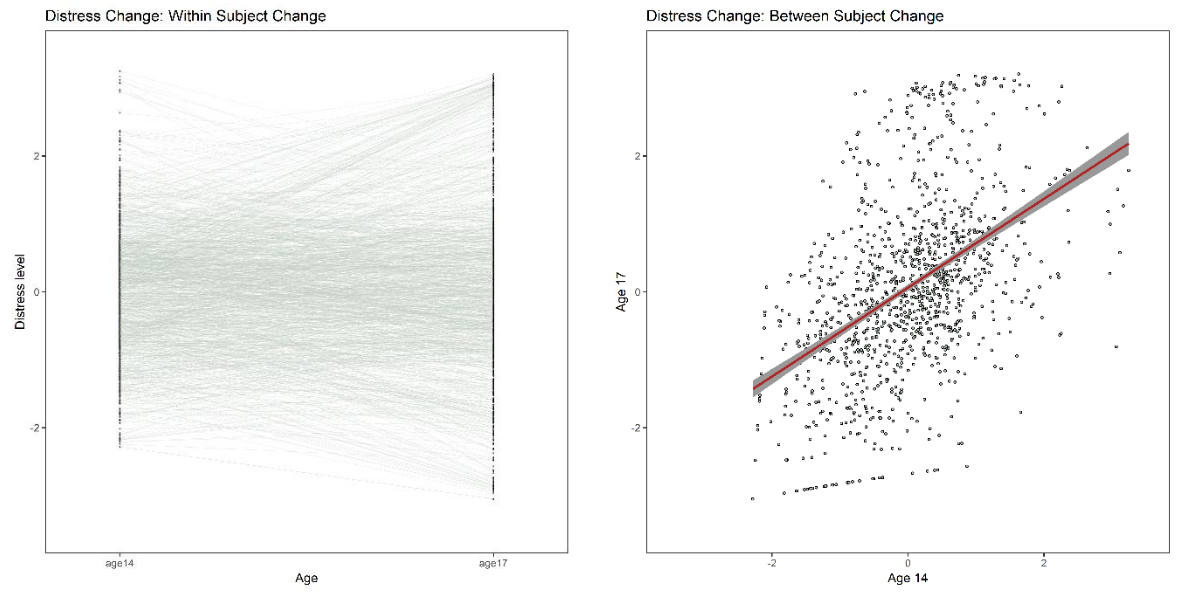

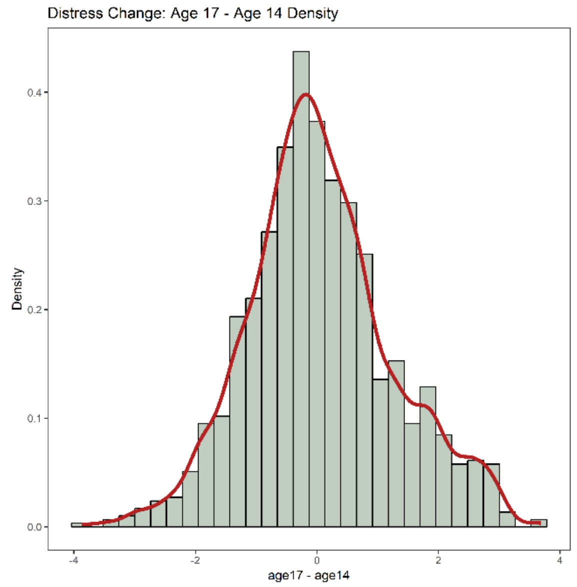

The upper left panel shows the within-subject (idiographic) change in distress from age 14 to age 17. Many adolescents have a rather stable trajectory, but others increase or decrease in distress. This largely variable pattern would also be expected in a naturalistic population sample. The upper right panel depicts the between-subject (nomothetic) association between age-14 and age-17 distress. The positive association indicates that on average adolescents with high age-14 distress also have high age-17 distress. The lower left panel shows that change in distress between age 14 and 17 (i.e. age-17 minus age-14 scores) is overall normally distributed. The plots indicate that there is variability in the change of distress that could be explained by resilience factors.

Supplement V

Variance inflation factors

**Mod**	**CA**	**gender**	**Frn**	**Fms**	**Fmc**	**Ngt**	**Pst**	**Brd**	**Rfl**	**Dst**	**Agg**	**Exp**	**D14**
B2	1.00	1.00											
M1	1.08	1.23	1.23	1.91	2.11	2.41	1.85	2.27	1.75	1.17	1.17	1.06	
M2	1.02	1.06											1.08
M3	1.08	1.24	1.27	1.91	2.13	3.03	1.87	2.49	1.76	1.17	1.21	1.06	3.52

Note. Mod = model; CA = childhood adversity; Frn = friendship support; Fms = family support; Fmc = family cohesion; Ngt = negative self-esteem; Pst = positive self-esteem; Brd = brooding; Rfl = reflection; Dst = distress tolerance; Agg = aggression; Exp = expressive suppression; D14 = age-14 distress; B2 = baseline model with CA and gender as predictors; M1 = main model with CA, gender and RFs as predictors; M2 = main model with CA, gender and age-14 distress as predictors; M3 = main model with CA, gender, RFs and age-14 distress as predictors. When taking the square root of the variance inflation factors, none is bigger than 2, which additionally underpins the absence of multicollinearity.

Supplement VI

Ordered categorical, or proportional odds models, have a "proportional odds" or also called "parallel slopes" assumption. This assumption necessitates that when the tested ordinal categories are dichotomized (e.g. here "a": low vs moderate and high, and "b": low and moderate vs high) the logistic prediction of the respective dichotomized categories results in two slopes (i.e. one for scenario "a" and one for scenario "b") that do not differ significantly from each other. If the slopes differ significantly, the proportional odds assumption does not hold and needs to be relaxed. The assumption can be determined for each predictor in the model and only for those predictors that do not meet the assumption separate slope values need to be estimated. This then results in a partial proportional odds model. It would also be possible to estimate a non-proportional odds model to circumvent the assumption for every variable in the model. However, this would be highly disadvantageous as it requires a vast amount of power. Hence we opted for the partial proportional odds model to ensure that we have as much power as possible. The below table depicts all the variables for which the proportional odds assumption was relaxed:

	**M1: RFs only**	**M2: D14 only**	**M3: RFs & D14**
**Whole sample**			
3-class models	-gender<br>-distress tolerance	-gender<br>-D14	-gender<br>-distress tolerance
3-class (models with reduced number of RFs)	-gender<br>-brooding	-	-gender
4-class model	-gender	-gender	-gender
**CA+ sample**			
3-class models	-gender	-gender<br>-D14	-gender<br>-D14
3-class (models with reduced number of RFs)	-gender<br>-brooding	-	-gender<br>-D14
4-class model	-gender	-gender<br>-D14	-gender<br>-D14
**CA- sample**			
3-class models	-gender	-gender	-gender<br>-distress tolerance
3-class (models with reduced number of RFs)	-gender	-	-gender
4-class model	-gender<br>-reflection	-gender	-gender<br>-reflection
**Female sample**			
3-class models	-distress tolerance	-D14	-distress tolerance
3-class (models with reduced number of RFs)	-distress tolerance	-	-distress tolerance
4-class model	X	-D14	-D14
**Male sample**			
3-class models	-friendship support<br>-aggression	-D14	-aggression<br>-D14
3-class (models with reduced number of RFs)	-negative self-esteem<br>-aggression	-	-aggression<br>-D14
4-class model	-reflection<br>-brooding	-D14	-reflection<br>-D14

Note. – means not tested. X means that all variables met the proportional odds assumption.

Supplement VII

The below tables depict the prediction accuracy of the prediction models described in the main manuscript. The first two tables depict subgroup accuracy comparisons for CA and gender models, respectively. The third table depicts accuracy comparisons for models including all RFs versus models that only include a subset of the RFs.

Subgroup accuracy comparisons for childhood adversity (CA) models

Model	Coefficient	CA+	CA-	Proportion test summary
M1 ordinal	Accuracy	54%	66%	Chi-squared = 3.8242, df = 1, p-value = 0.051
	Correct predictions	84	82	
	Total predictions	156	124	
M2 ordinal	Accuracy	60%	69%	Chi-squared = 2.1104, df = 1, p-value = 0.146
	Correct predictions	94	86	
	Total predictions	156	124	
M3 ordinal	Accuracy	58%	69%	Chi-squared = 2.6657, df = 1, p-value = 0.103
	Correct predictions	91	85	
	Total predictions	156	124	
M1 linear	Accuracy	34.62%	40.32%	Chi-squared = 0.73489, df = 1, p-value = 0.391
	Correct predictions	54	50	
	Total predictions	156	124	
M2 linear	Accuracy	32.69%	38.71%	Chi-squared = 0.84703, df = 1, p-value = 0.357
	Correct predictions	51	48	
	Total predictions	156	124	
M3 linear	Accuracy	36.54%	38.71%	Chi-squared = 0.06176, df = 1, p-value = 0.804
	Correct predictions	57	48	
	Total predictions	156	124	
M1 ordinal 4 classes	Accuracy	47%	51%	Chi-squared = 0.29897, df = 1, p-value = 0.585
	Correct predictions	73	63	
	Total predictions	156	124	
M2 ordinal 4 classes	Accuracy	56%	57%	Chi-squared = 7.0965e-31, df = 1, p-value = 1
	Correct predictions	87	70	
	Total predictions	156	124	
M3 ordinal 4 classes	Accuracy	48%	54%	Chi-squared = 0.75649, df = 1, p-value = 0.384
	Correct predictions	75	67	
	Total predictions	156	124	

Note. M1 = Model 1 contains the ten RFs, M2 = Model 2 contains age-14 distress, M3 = Model 3 contains both the RFs and age-14 distress as predictors for age-17 distress. Correct predictions = number of correctly predicted adolescents, Total predictions = number of adolescents that could have been predicted correctly, Accuracy = ratio correct predictions divided by total predictions. df = degrees of freedom.

Subgroup accuracy comparisons for gender models

Model	Coefficient	female	male	Proportion test summary
M1 ordinal	Accuracy	58%	61%	Chi-squared = 0.29082, df = 1, p-value = 0.590
	Correct predictions	88	78	
	Total predictions	153	127	
M2 ordinal	Accuracy	59%	64%	Chi-squared = 0.52365, df = 1, p-value = 0.469
	Correct predictions	90	81	
	Total predictions	153	127	
M3 ordinal	Accuracy	58%	61%	Chi-squared = 0.18409, df = 1, p-value = 0.668
	Correct predictions	89	78	
	Total predictions	153	127	
M1 linear	Accuracy	32.90%	38.89%	Chi-squared = 0.83392, df = 1, p-value = 0.361
	Correct predictions	50	49	

	Total predictions	152	126	
M2 linear	Accuracy	35.53%	41.27%	Chi-squared = 0.73526, df = 1, p-value = 0.391
	Correct predictions	54	52	
	Total predictions	152	126	
M3 linear	Accuracy	34.87%	42.06%	Chi-squared = 1.2222, df = 1, p-value = 0.269
	Correct predictions	53	53	
	Total predictions	152	126	
M1 ordinal 4 classes	Accuracy	43%	48%	Chi-squared = 0.4571, df = 1, p-value = 0.499
	Correct predictions	66	60	
	Total predictions	154	126	
M2 ordinal 4 classes	Accuracy	53%	53%	Chi-squared = 1.6826e-30, df = 1, p-value = 1
	Correct predictions	81	67	
	Total predictions	154	126	
M3 ordinal 4 classes	Accuracy	49%	52%	Chi-squared = 0.14779, df = 1, p-value = 0.701
	Correct predictions	76	66	
	Total predictions	154	126	

Note. M1 = Model 1 contains the ten RFs, M2 = Model 2 contains age-14 distress, M3 = Model 3 contains both the RFs and age-14 distress as predictors for age-17 distress. Correct predictions = number of correctly predicted adolescents, Total predictions = number of adolescents that could have been predicted correctly, Accuracy = ratio correct predictions divided by total predictions. df = degrees of freedom.

Accuracy comparison for models including all RFs versus those including a subset of the RFs

**Model**	**Coefficient**	**All RFs**	**3 RFs**	**Proportion test summary**
M1 ordinal	Accuracy	64%	62%	Chi-squared = 0.374, df = 1, p-value = 0.541
	Correct predictions	181	173	
	Total predictions	281	281	
M3 ordinal	Accuracy	63%	60%	Chi-squared = 0.6091, df = 1, p-value = 0.435
	Correct predictions	178	168	
	Total predictions	281	281	
M1 linear	Accuracy	37.14%	37.14%	Chi-squared = 0, df = 1, p-value = 1
	Correct predictions	104	104	
	Total predictions	280	280	
M3 linear	Accuracy	40.71%	40.71%	Chi-squared = 0, df = 1, p-value = 1
	Correct predictions	114	114	
	Total predictions	280	280	

Note. M1 = Model 1 contains the ten RFs, M2 = Model 2 contains age-14 distress, M3 = Model 3 contains both the RFs and age-14 distress as predictors for age-17 distress. Correct predictions = number of correctly predicted adolescents, Total predictions = number of adolescents that could have been predicted correctly, Accuracy = ratio correct predictions divided by total predictions. df = degrees of freedom.

Supplement VIII

For the CA+ group we tested six RFs in addition to gender, as those were significant in the multivariable model, namely: friendship support, family cohesion, positive self-esteem, brooding, distress tolerance, and aggression. Those models were similarly predictive as the models with all 10 RFs and gender (change in accuracy: ordinal models from 54% to 58%; linear models from 34.62% to 33.97%). We also tested those two models while additionally including age-14 distress, which were again similar as the models with gender, the 10 RFs and age-14 distress (change in accuracy: ordinal models from 58% to 60%; linear models from 36.54% to 35.90%).

For the CA- group we tested four RFs in addition to gender, as those were significant in the multivariable model, namely: family support, positive self-esteem, negative self-esteem, and brooding. Those models were similarly predictive as the models with all 10 RFs and gender

(change in accuracy: ordinal models from 66% to 65%; linear models from 40.32% to 41.94%). We also tested those two models while additionally including age-14 distress, which were again similar as the models with gender, the 10 RFs and age-14 distress (change in accuracy: ordinal models from 69% to 68%; linear models from 38.71% to 37.10%).

For female adolescents we tested three RFs, as those were significant in the multivariable model, namely: negative self-esteem, brooding and distress tolerance. Those models were similarly predictive as the models with all 10 RFs and CA (change in accuracy: ordinal models from 58% to 56%; linear models from 32.90% to 31.58%). We also tested those two models while additionally including age-14 distress, which were again similar as the models with CA, the 10 RFs and age-14 distress (change in accuracy: ordinal models from 58% to 58%; linear models from 34.87% to 36.84%).

For male adolescents we tested three RFs, as those were significant in the multivariable model, namely: positive self-esteem, negative self-esteem and aggression. Those models were similarly predictive as the models with all 10 RFs and CA (change in accuracy: ordinal models from 61% to 61%; linear models from 38.89% to 43.65%). We also tested those two models while additionally including age-14 distress, which were again similar as the models with CA, 10 RFs and age-14 distress (change in accuracy: ordinal models from 61% to 61%; linear models from 42.06% to 38.89%).

Here we did not test whether the accuracy differed significantly between the subgroups (i.e. CA+ vs CA-, and female vs male) as we tested the subgroups with different sets of RF predictors.

Supplement IX

Similar to the 3-class model, the 4-class model revealed a plausible distress severity solution, split in a low, low/moderate, moderate/high and a high distress severity class. We also conducted three *ordinal* prediction models with the four-class distress variable as ordered categorical outcome variable. Of the three models one again contained the RFs (M1), one age-14 distress (M2) and one both (RFs and age-14 distress; M3) in addition to gender and CA. The three models had a low accuracy ranging from 50% to 52% (see Table below), resulting for all three models in about 1 in 2 adolescents who were predicted into their correct distress severity class. The results were comparable when we split the adolescents into CA+ (accuracy: M1 = 47%, M2 = 56%, M3 = 48%), CA- (accuracy: M1 = 51%, M2 = 57%, M3 = 54%), female (accuracy: M1 = 43%, M2 = 53%, M3 = 49%) and male groups (accuracy: M1 = 48%, M2 = 53%, M3 = 52%). Moreover, the prediction accuracy did not differ significantly between the CA and gender subgroups (see Supplement VII).

Ordinal prediction analyses for the whole group: for RFs only (M1), age-14 distress only (M2), and RFs and age-14 distress together (M3)

	**M1: RFs only**		**M2: D14 only**		**M3: RFs & D14**	
	**observed**	**predicted**	**observed**	**predicted**	**observed**	**predicted**
Residual deviance	1903.68	-	1849.99	-	1836.02	-
ROC	-	low=0.70<br>l/m=0.62<br>m/h=0.69<br>high=0.68	-	low=0.71<br>l/m=0.63<br>m/h=0.70<br>high=0.71	-	low=0.71<br>l/m=0.61<br>m/h=0.69<br>high=0.70
Sensitivity	-	low=0.79<br>l/m=0.46<br>m/h=0.07<br>high=0.04	-	low=0.74<br>l/m=0.58<br>m/h=0.10<br>high=0.04	-	low=0.71<br>l/m=0.56<br>m/h=0.12<br>high=0.07

Specificity	-	low=0.61<br>l/m=0.65<br>m/h=0.97<br>high=0.98	-	low=0.69<br>l/m=0.62<br>m/h=0.98<br>high=0.97	-	low=0.67<br>l/m=0.62<br>m/h=0.98<br>high=0.97
Accuracy	-	0.50<br>low=0.70<br>l/m=0.56<br>m/h=0.52<br>high=0.51	-	0.52<br>low=0.71<br>l/m=0.60<br>m/h=0.54<br>high=0.51	-	0.51<br>low=0.69<br>l/m=0.59<br>m/h=0.55<br>high=0.52
Low distress severity	116	155 of which<br>- 91 correct<br>- 46 false l/m<br>- 09 false m/h<br>- 09 false high	116	138 of which<br>- 86 correct<br>- 35 false l/m<br>- 05 false m/h<br>- 12 false high	116	137 of which<br>- 82 correct<br>- 37 false l/m<br>- 07 false m/h<br>- 11 false high
Low/mod severity	96	108 of which<br>- 44 correct<br>- 24 false low<br>- 26 false h/m<br>- 14 false high	96	126 of which<br>- 56 correct<br>- 27 false low<br>- 30 false h/m<br>- 13 false high	96	124 of which<br>- 54 correct<br>- 32 false low<br>- 25 false h/m<br>- 13 false high
Mod/high severity	42	11 of which<br>- 03 correct<br>- 00 false low<br>- 05 false l/m<br>- 03 false high	42	9 of which<br>- 04 correct<br>- 01 false low<br>- 03 false l/m<br>- 01 false high	42	10 of which<br>- 05 correct<br>- 01 false low<br>- 03 false l/m<br>- 01 false high
High distress severity	27	7 of which<br>- 01 correct<br>- 01 false low<br>- 01 false l/m<br>- 04 false m/h	27	8 of which<br>- 01 correct<br>- 02 false low<br>- 02 false l/m<br>- 03 false m/h	27	10 of which<br>- 02 correct<br>- 01 false low<br>- 02 false l/m<br>- 05 false m/h

Note. D14 = age-14 distress. All models were computed with childhood adversity and gender as predictors. ROC = receiver operating characteristic. Accuracy = relative number of correctly predicted cases. Sensitivity = e.g. for low distress: the number of adolescents who are correctly predicted into the low distress group divided by all adolescent who are actually in the low distress group. Specificity = e.g. for low distress: the number of adolescents who are correctly not predicted into the low distress group divided by all adolescent who are actually not in the low distress group. Variable for which the proportional odds assumption was relaxed can be found in Supplement VI.

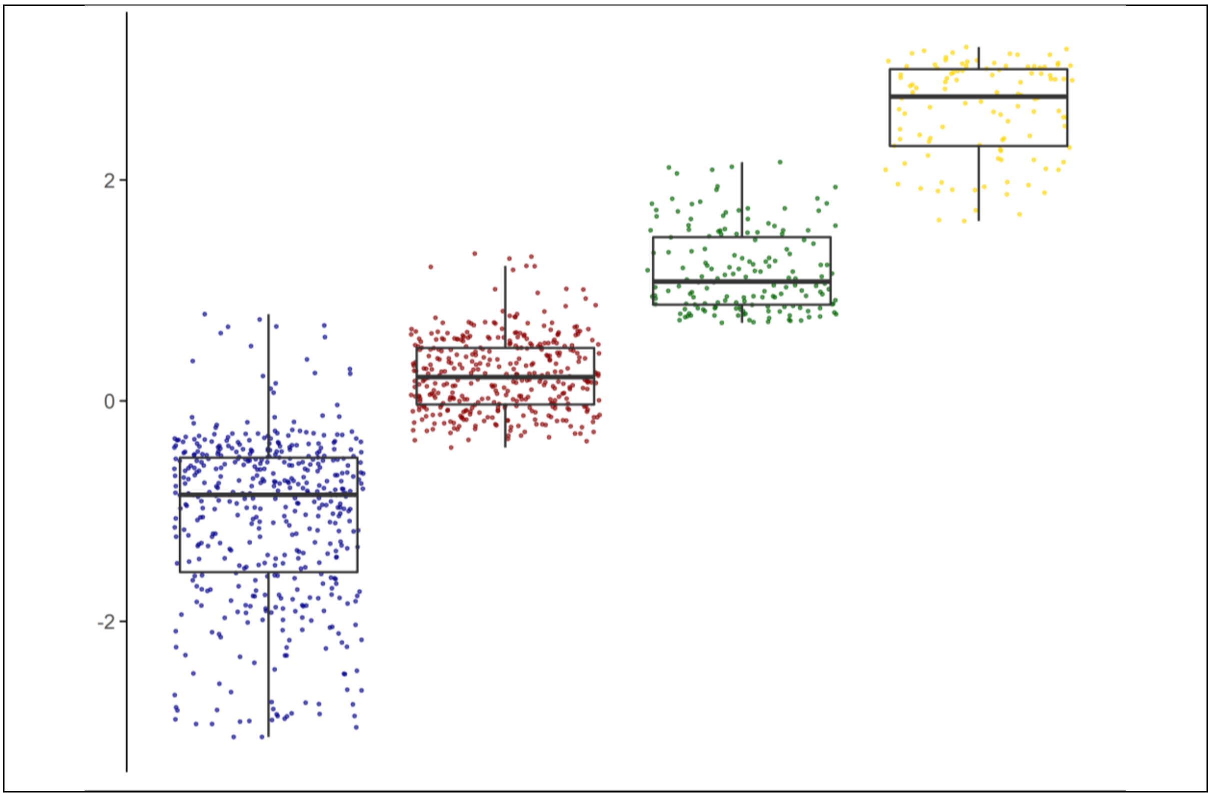

Figure 1. Four-class distress solution (low: n = 466; low/moderate: n = 386; moderate/high: n = 168; high = 110) plotted against the continuous distress severity scores. Center line = median (50% quantile); lower box

limit =25% quantile; upper box limit = 75% quantile; lower whisker = smallest observation greater than or equal to the lower box limit -1.5 x Inter Quartile Range (IQR); upper whisker = largest observation less than or equal to the upper box limit + 1.5 x Inter Quartile Range (IQR).

REFERENCES

1. Goodyer IM, Wright C, Altham PME. Recent friendships in anxious and depressed school age children. *Psychol Med*. 1989;19(1):165-174. doi:10.1017/S0033291700011119

2. Epstein NB, Baldwin LM, Bishop DS. The McMaster Family Assessment Device. *J Marital Fam Ther*. 1983;9(2):171-180.

3. Rosenberg M. *Society and the Adolescent Self-Image*. Princeton, NJ: Princeton University Press; 1965.

4. Treynor W, Gonzalez R, Nolen-Hoeksema S. Rumination reconsidered: A psychometric analysis. *Cognit Ther Res*. 2003;27(3):247-259.

5. Bould H, Joinson C, Sterne J, Araya R. The Emotionality Activity Sociability Temperament Survey: Factor analysis and temporal stability in a longitudinal cohort. *Pers Individ Dif*. 2013;54:628-633. doi:10.1016/j.paid.2012.11.010

6. Goodyer IM, Tsancheva S, Byford S, et al. Improving mood with psychoanalytic and cognitive therapies (IMPACT): a pragmatic effectiveness superiority trial to investigate whether specialised psychological treatment reduces the risk for relapse in adolescents with moderate to severe unipolar dep... *Trials*. 2011;12:175. doi:10.1186/1745-6215-12-175

7. Messer SC, Angold A, Costello EJ. Development of a Short Questionnaire for Use in Epidemiological Studies of Depression in Children and Adolescents: Factor Composition and Structure across Development. *Int J Methods Psychiatr Res*. 1995;5:251-262.

8. Reynolds CR, Richmond BO. What I Think and Feel: A Revised Measure of Children's Manifest Anxiety. *J Abnorm Child Psychol*. 1978;6(2):271-280.

9. Clark SL, Muthén B, Kaprio J, D'Onofrio BM, Viken R, Rose RJ. Models and Strategies for Factor Mixture Analysis: An Example Concerning the Structure Underlying Psychological Disorders. *Struct Equ Model A Multidiscip J*. 2013;20(4):681-703. doi:10.1080/10705511.2013.824786

10. Kampstra P. Beanplot: A Boxplot Alternative for Visual Comparison of Distributions. *J Stat Softw*. 2008;28(1):1-9. http://www.jstatsoft.org/v28/c01/.

11. Schlegel B, Steenbergen M. brant: Test for Parallel Regression Assumption. R package version 0.2-0. 2018. https://cran.r-project.org/package=brant.

12. Fox J, Weisberg S. *An {R} Companion to Applied Regression (Second Edition).* CA: Thousand Oaks: Sage.; 2011.

13. Kuhn M. caret: Classification and Regression Training. 2018. https://cran.r-project.org/package=caret/.

14. Hothorn T, Hornik K, van de Wiel MA, Zeileis A. Implementing a Class of Permutation Tests: The coin Package. *J Stat Softw*. 2008;28(8):1-23. http://www.jstatsoft.org/v28/i08/.

15. Wickham H, François R, Henry L, Müller K. dplyr: A Grammar of Data Manipulation. R package version 0.7.7. https://cran.r-project.org/package=dplyr. Published 2018.

16. R Core Team. foreign: Read Data Stored by “Minitab”, “S”, “SAS”, “SPSS”, “Stata”, “Systat”, “Weka”, “dBase”, ... R package version 0.8-70. 2017. https://cran.r-project.org/package=foreign.

17. Harrell Jr. FE. Hmisc: Harrell Miscellaneous. 2018. https://cran.r-project.org/package=Hmisc.

18. Venables WN, Ripley BD. *Modern Applied Statistics with S (Fourth Edition)*. New York: Springer; 2002.

19. van Buuren S, Groothuis-Oudshoorn K. mice: Multivariate Imputation by Chained Equations in R. *J Stat Softw*. 2011;45(3):1-67. http://www.jstatsoft.org/.

20. Yan Y. MLmetrics: Machine Learning Evaluation Metrics. 2016. https://cran.r-project.org/package=MLmetrics.

21. Grosjean P, Ibanez F. pastecs: Package for Analysis of Space-Time Ecological Series. R package version 1.3.21. https://cran.r-project.org/package=pastecs. Published 2018.

22. Robin X, Turck N, Hainard A, et al. pROC: An open-source package for R and S+ to analyze and compare ROC curves. *BMC Bioinformatics*. 2011;12:77. doi:10.1186/1471-2105-12-77

23. Epskamp S, Cramer AOJ, Waldorp LJ, Schmittmann VD, Borsboom D. qgraph: Network Visualizations of Relationships in Psychometric Data. *J Stat Softw*. 2012;48(4):1-18. http://www.jstatsoft.org/.

24. Groemping U. Relative Importance for Linear Regression in R: The Package relaimpo. *J Stat Softw*. 2006;17(1):1-27. http://www.jstatsoft.org/.

25. Wickham H. Reshaping data with the reshape package. *J Stat Softw*. 2007;21(12):2007.

26. Jorgensen TD, Pornprasertmanit S, Schoemann AM, Rosseel Y. semTools: Useful tools for structural equation modeling. R package version 0.5-1.933. https://cran.r-project.org/package=semTools. Published 2018.

27. Yee TW. The VGAM Package for Categorical Data Analysis. *J Stat Softw*. 2010;32(10):1-34. http://www.jstatsoft.org/v32/i10/.
