# Supplementary material for: Embracing the positive: an examination of how well resilience factors at age 14 can predict distress at age 17
Source: Transl Psychiatry. 2020 Aug 5;10:272. doi: 10.1038/s41398-020-00944-w (PMC7406495; doi:10.1038/s41398-020-00944-w)
Supplement: Supplementary file 2 — Supplementry information2 [file 41398_2020_944_MOESM2_ESM.pdf]

Additional file containing the Manuscript
“Embracing the positive:
An examination of how well resilience factors at age 14 can predict distress at age 17”
based on non-imputed data

J. Fritz,[1,*] J. Stochl,[1,2] I.M. Goodyer,[1] A.-L. van Harmelen,[1,A] & P.O. Wilkinson[1,A]

* Correspondence: Jessica Fritz, jf585@cam.ac.uk
[1] University of Cambridge, Department of Psychiatry, United Kingdom
[2] Charles University, Department of Kinanthropology, Czech Republic
[A] shared last author

One-in-two people suffering from mental health problems develop such distress before or during adolescence. Research has shown that distress can predict itself well over time. Yet, little is known about how well resilience factors (RFs), i.e. those factors that decrease mental health problems, predict subsequent distress. Therefore, we investigated which RFs are the best indicators for subsequent distress and with what accuracy RFs predict subsequent distress. We examined three *inter*-personal (e.g. friendships) and seven *intra*-personal RFs (e.g. self-esteem) and distress in 1130 adolescents, at age 14 and 17. We estimated the RFs and a continuous distress-index using factor analyses, and ordinal distress-classes using factor mixture models. We then examined how well age-14 RFs and age-14 distress predict age-17 distress, using stepwise linear regressions, relative importance analyses, as well as ordinal and linear prediction models. Low brooding and low negative self-esteem RFs were the most important indicators for age-17 distress. RFs and age-14 distress predicted age-17 distress similarly. The accuracy was acceptable for ordinal (low/moderate/high age-17 distress-classes: 63-66%), but low for linear models (39-47%). Crucially, the accuracy remained similar when only negative self-esteem and brooding RFs were used instead of all 10 RFs (ordinal=61%; linear=38%); correctly predicting for about two-in-three adolescents whether they have low, moderate or high distress three years later. RFs, and particularly brooding and negative self-esteem, seem to predict subsequent distress similarly well as distress can predict itself. As assessing brooding and negative self-esteem can be strength-focussed and is time-efficient, those RFs may be promising for risk-detection and translational intervention research.

Keywords: resilience factors, protective factors, distress, psychopathology, mental health promotion, prediction, adolescence

Words: 4507 (+ 1557 words in the methods section)

Every year, about 1 in 5 people experience mental disorders,[1,2] of which the most prevalent mental illnesses are depressive and anxiety disorders.[1] Half of such mental illnesses first emerge during adolescence.[3] About 1 in 3 adolescents have an episode of an anxiety disorder and more than 1 in 10 an episode of a mood disorder, between the ages of 13 and 18.[4] The prevalence of anxiety disorders tends to remain stable during adolescence, however, mood disorders double between the ages of 13 and 18.[4] Hence, adolescence seems to be a particularly sensitive time period for the emergence of mental health problems and it is therefore imperative to characterize and predict such vulnerability to psychopathological distress properly.

A growing number of studies has developed screening tools and risk prediction models – also known as risk calculators – for mental health problems.[5,6] For example, Dinga and colleagues (2018)[7] have shown that, among a large variety of psychological and biological variables, only mood severity predicted subsequent depressive symptomology significantly.[7] Still, their prediction model revealed an acceptable accuracy.[7] Similarly, Lewis and colleagues (2019) have shown that a constellation of demographics, psychopathology symptoms (i.e. psychotic and internalizing symptoms), and adversity variables can together satisfactorily predict whether adolescents develop post-traumatic stress disorder, following trauma exposure.[8] In a recent systematic review, summarizing literature on mental health screening tools and risk models, 60 studies were identified for depression related diagnoses, 13 for psychopathological stress, five for anxiety related diagnoses, and five for well-being.[6] Importantly, the majority of those studies used symptom-related (e.g. questionnaires and interviews), demographical (e.g. adverse life-events), or biological indicators (e.g. inflammatory markers, cortisol, metabolic syndrome, brain-derived neurotrophic factor, white and grey matter, and heart rate variables).[5–7,9] Thus, previous studies primarily examined predictors that are relatively static (e.g. ethnicity or gray matter) and/or risk factors that increase the development of mental health problems (e.g. negative life-events or prior psychiatric symptoms). Focussing on static and risk factors, however, is only half the story, as it fails to address factors that are amenable and promote mental health. The resilience literature has already identified various factors that are associated with improved subsequent mental health,[10–14] which seem to be overlooked in the development of screening tools and risk calculators. A notable exception is the study of Chen and colleagues (2015)[15] in which self-esteem was used to predict subsequent anxiety. Another important exception is the recent study of Meehan and colleagues (2020)[16], which included alongside various risk indicators four potential resilience factors (sibling warmth, adult involvement, social cohesion and status among peers), to predict internalizing and externalizing disorders following victimization.

Here, we aim to extend the existing literature in several ways. Firstly, we use resilience factors (RFs) as predictors, i.e. factors that have been found to reduce the risk of psychological distress following adverse experiences.[17] We derived the RFs that we study here from a preregistered systematic review,[17] in which RFs were defined as those factors that moderate and/or mediate the relationship between childhood adversity and subsequent mental health problems. In the resilience literature there is a sparse but ongoing discourse about whether resilience and risk factors are opposing sides of the same coin (for a detailed discussion see Fritz, Stochl and colleagues[18]). Some RFs and risk factors seem indeed to be on opposing sides of the same continuum (e.g. RF = high friendship support & risk factor = low friendship support)[19], whereas for others this apparent dichotomy seems more complex. For example, *high* rumination can be both an RF and a risk factor depending whether its content is positive or negative (e.g. RF = *high positive* rumination & risk factor = *low positive* ruminations; RF = *low negative* rumination & risk factor = *high negative* rumination; while *high positive* and *high negative* rumination often go together[20]). Importantly, regardless of whether resilience and risk factors operate on the same continuum, studying the predictive value of RFs has universal appeal as it focuses on what promotes good mental health rather than on what increases mental

health problems.[18] Secondly, we extend the existing literature through focusing exclusively on factors that are amenable to psychotherapeutic change, which is in contrast to the majority of the above reviewed studies, as those mainly focused on relatively static demographic (e.g. ethnicity) and biological (e.g. grey or white matter volume) predictors. More specifically, we predict psychopathological distress from 10 amenable RFs. Three of those RFs operate on an *inter*-individual level: friendship support, family support and family cohesion; and seven on an *intra*-individual level: high positive self-esteem, low negative self-esteem, low brooding, low ruminative reflection, high distress tolerance, a low aggression potential and low expressive suppression.[17] Importantly, all those RFs on their own have been found to decrease subsequent mental health problems, yet, research investigating multiple RFs at the same time is so far scarce.[21–23] Recently, we found that these RFs reduce concurrent psychopathological distress with a similar degree in adolescents with and without prior exposure to adversity. Moreover, we have shown that the RFs interrelate strongly and can be described as a complex interacting system.[18] This supports the notion that models that succeed in taking all those factors into account may ecologically be more valid and may successfully reveal those RFs that are particularly important in reducing the risk of mental health problems.

Recently, research has also shed light on the benefits of describing mental health problems as distress continua rather than as discrete diagnosis specific constructs. For example, several studies show that modelling psychopathological symptoms as a continuous latent factor captures a wide range of mental health symptomatology, in terms of both severity and breadth of symptomatology,[24–28] and even seems to generalize well to other disorders.[25] Therefore, such latent continuous constructs may be particularly informative for transdiagnostic prevention and intervention research. Moreover, hybrid models have been developed that describe mental health symptoms as a continuous latent factor and then add categorical classes to the latent factor that differentiate between subgroups on the latent mental distress continuum (e.g. as defined by differences in the distress severity).[29] Categorical distress scores derived from those models may be particular useful for prediction purposes, as they allow for the estimation of predictive sensitivity and specificity, while taking into account the continuous nature of distress. Yet, to the best of our knowledge, transdiagnostic distress indices have so far rarely been used for predictive purposes and is therefore the third way in which we extend the existing literature.

In sum, we aim to extend the existing literature (a) by using resilience factors rather than risk markers as predictors for subsequent psychopathology, (b) by using amenable (i.e. social, emotional, cognitive and behavioural) rather than static variables (e.g. ethnicity or biological predispositions) as predictors, and (c) by using transdiagnostic distress indices rather than discrete diagnosis specific variables as outcome variables. To this end, we use data from the ROOTS population cohort (n = 1130)[30] to predict distress at age 17 from RFs assessed at age 14, covering the adolescent period during which about half of all mental illnesses start emerging. Given the powerful predictive effects of past mental distress, we evaluate in addition to the relative effects of RFs also the relative effect of distress at age 14 when predicting distress at age 17. A cascade of studies has shown that childhood adversity (CA) vastly increases the risk for mental health problems during adolescence and adulthood.[31–34] Therefore, throughout all analyses, we take the effect of CA before the age of 14 into account. Additionally, we control for gender effects, as being female has frequently been found to increase the risk for distress.[e.g. 26] In sum, we aim to examine:

a) to what degree RFs can explain subsequent distress,
b) which RFs are the best indicators for subsequent distress, and
c) with what accuracy RFs can predict distress levels three years later.

METHODS

Sample

The ROOTS study is a population cohort for which 1238 adolescents were recruited at age 14 and reassessed at age 17. The adolescents were recruited in 2005 and 2006, via 18 schools in and around Cambridgeshire. The adolescents and one parent had to provide written informed consent. ROOTS was approved by the Cambridgeshire Research Ethics Committee (03/302) and was conducted along the lines of Good Clinical Practice guidelines and the Declaration of Helsinki.[30]

Participants

Here we included adolescents who had complete data on all RFs at age 14 as well as distress at age 14 and 17, and had information on gender and the presence/absence of adverse experiences before the age of 14 (N = 729).

RFs

In accordance with Fritz et al. 2018[23] and 2019[18], we investigated 10 RFs that were identified in our preregistered systematic review[17] and were assessed in ROOTS[30]. All RFs were assessed at age 14:

1. Friendship support: five items of the Cambridge Friendships Questionnaire.[35]
2. Family support: five items of the McMaster Family Assessment Device.[36]
3. Family cohesion/climate: seven items of the McMaster Family Assessment Device. For brevity we write family cohesion throughout the manuscript.[36]
4. Positive self-esteem: five items of the Rosenberg self-esteem scale.[37]
5. Negative self-esteem: five remaining items of the Rosenberg self-esteem scale (of note, the items are reversed).[37]
6. Reflective rumination: five items of the Ruminative Response Scale (RRS; of note, the items are reversed).[38,39]
7. Ruminative brooding: five items of the RRS (of note, the items are reversed).[38,39]
8. Aggression: four items of the Behaviour Checklist (11 questions based on the DSM-IV criteria for conduct problems; of note, the items are reversed).[40,41]
9. Distress tolerance: five items of the Emotionality Activity Sociability Temperament Survey.[42]
10. Expressive suppression*:* one item of the Antisocial Process Screening Device (of note, the item is reversed).[43]

Items of five RFs had to be reversed to ensure that all RFs are scored in such a way that high values are protective. The first eight RFs are based on self-report, and the last two on parent report. The psychometrics of the RF measures are described in Supplement XIII in Fritz, Fried and colleagues.[23]

Distress

At age 14 and 17, distress was assessed with 41 items of which 28 had a focus on anxiety symptoms (Revised Children's Manifest Anxiety Scale[44]) and 13 a focus on depressive symptoms (Short Mood and Feelings Questionnaire[45]).

Childhood Adversity

CA was assessed with the Cambridge Early Experiences Interview (CAMEEI), which is a semi-structured interview performed with the primary carer.[46] CAs were defined as adverse experiences or severely stressful events that happened between birth and the age of 14. The assessed CAs include a wide range of intra-family events/experiences (e.g. sexual, physical or emotional maltreatments, or parental mental illness), but also cover external events (e.g. a fire or exposure to war). For a detailed description see Dunn and colleagues.[46] These authors clustered the adolescents based on their CA experiences into four latent classes (i.e. no, moderate, severe and atypical parenting CA), separately for the time periods early (age 0 to 5), middle (age 5 to 11) and late childhood (age 11 to 14).[46] As in previous reports on this sample,[23] we dichotomized the CA variable in CA+, which is 'moderate, severe and/or atypical parenting CA' for at least one of the three time periods, and CA-, which is 'no CA' for any of the three time periods.

Analyses

Variable estimation. Prior to the main analyses we computed the RFs based on unidimensional confirmatory factor analyses (CFAs; except for expressive suppression as this was assessed with only one item). We use factor scores and not sum scores to evade tau-equivalence and to decrease measurement error as much as possible (for a rationale and explanation see Additional file V Part A in Fritz, Stochl et al., 2019[18]). As all items ranged between three and six answer categories, we used categorical CFAs with a weighted least square mean and variance adjusted (WLSMV) estimator. The distress factor was similarly estimated using a longitudinal, unidimensional, categorical CFA (also with the WLSMV estimator), and was identified according to the strongly invariant model described by Wu and Estabrook (for a more detailed rationale see Supplement I).[47]

Prediction analyses. First, we performed a series of multiple linear regressions to predict distress at age 17. The first two models functioned as baseline models, one only included CA (model B1) and the other one included CA and gender as regressors (model B2). The next three models were the main models of interest: All contained CA and gender as regressor, the first model additionally contained the ten RFs (model M1), the second model additionally contained distress at age 14 (model M2), and the third model additionally contained both the RFs and distress at age 14 (model M3). Those analyses were performed to examine the directionality of the regressors (i.e. +/- sign of the b-values) and to investigate which regressors add significant variance to the explanation of distress at age 17. We additionally compared the models against each other using Likelihood-Ratio tests. Moreover, we re-estimated the models separately for the CA+ and the CA- groups as well as for males and females, to explore group effects.

Second, we aimed to disentangle the relative importance (RI) of the regressors in explaining general distress at age 17. Disentangling the RIs is of particular importance when the regressors are (or are assumed to be) strongly correlated, as every order of regressors then results in a different decomposition of sum of squares.[48] Here, we examined the RI metric "lmg" (cf. Lindeman, Merenda and Gold),[49] which calculates sequential R^2s while permuting and then averaging over the regressor orders.[48] To this end, we performed the three above described main models (M1, M2, and M3) as RI analyses. Moreover, we repeated the analyses separately for the CA+ and the CA- group as well as for males and females, to investigate differences in result patterns between subgroups.

Third, we conducted prediction analyses, to test with what accuracy the RFs and general distress at age 14 predict distress at age 17. We again used the three main models described

above (M1, M2 and M3). All three prediction models were conducted once as a *categorical* model, with general distress at age 17 as categorical outcome variable, and once as *linear* models, with general distress at age 17 as a continuous outcome variable. For the categorical distress variable we conducted a series of factor mixture models,[29] which are hybrid models that add latent classes on top of the latent factors, with different invariance levels between the classes. We did this to classify the adolescents based on their distress profiles into categorical distress classes, while also taking into account the continuous nature of distress. Firstly, we applied latent class analyses to identify possible class solutions and then conducted one-factor mixture models with the appropriate class solutions (factor mixture model analyses details can be found in Supplement II). For a factor mixture model solution with two classes we planned to use *logistic* prediction models, for a factor mixture model solution with three or more unordered classes we planned to use *multinomial* prediction models, and for a factor mixture model solution with three or more ordered classes we planned to use *ordinal* prediction models. For the prediction analyses the sample was quasi-randomly split into a training sample (75%; n = ~545) and a testing sample (25%; n = ~180; quasi-randomly means that that the relative class proportion of age-17 distress was kept equal between the training and the testing sample). We chose to have a larger training than testing sample, to be able to estimate as accurate prediction models as possible, particularly given that categorical prediction models require a substantial amount of power (relatively more than linear models, depending on the category number and size of the outcome variable). To determine the best link function for the categorical prediction models (i.e. logistic or probit) we used the Akaike Information Criterion (AIC) and the residual deviance as model comparison indices. We then used the models resulting from the training procedures to predict distress at age 17 in the testing sample. To evaluate *categorical* prediction models, we calculated the amount of predicted distress scores that were predicted into their observed distress class. To evaluate the *linear* prediction models, we used the standard errors (SEs) of the age-17 distress factor scores and computed person-specific 95% confidence intervals (CI). We then calculated for how many adolescents our model could predict distress scores that fell into their respective 95% factor score CI. We again, also computed the analyses separately for the CA+ and the CA- group as well as for males and females, to investigate differences in result patterns between subgroups. This time, we could quantify the differences between the CA and the gender subgroups using proportion comparison tests, as we could describe the determined accuracies as accuracy proportions.

Software. Most analyses were performed in R version 3.5.1 (R packages are reported in Supplement III).[50] The factor scores and SEs for age-14 and age-17 distress were estimated in MPlus 8.2,[51] as it was not possible to compute the SEs based on categorical data in R. Similarly, we performed the latent class and factor mixture model analyses in MPlus as this allowed us to specify the items as categorical.[51]

Data availability. Data for this specific paper has been uploaded to the Cambridge Data Repository https://doi.org/10.17863/CAM.46642 and is password protected. Our participants did not give informed consent for their measures to be made publicly available, and it is possible that they could be identified from this data set. Access to the data supporting the analyses presented in this paper will be made available to researchers with a reasonable request to openNSPN@medschl.cam.ac.uk.

Code availability. Analysis code is available from http://jessica-fritz.com/.

RESULTS

Sample

After excluding four adolescents who qualified as outliers in the multivariate space, we could include 725 adolescents of which 377 were exposed (CA+) and 348 were not exposed to prior CA (CA-; see Table 1). The CA groups did not differ in age or gender proportions. SES was higher and a prior psychiatric history was less likely in the CA- than in the CA+ group. Of the 725 participants, 415 were female and 310 male. The male and the female groups did neither differ in age nor SES. Female adolescents were more likely to have a prior psychiatric history.

Table 1

Sample description, split for CA and gender

	**CA+ (n = 377)**	**CA- (n = 348)**	**χ^2 /z / t**	**p-value**
gender	Female = 226<br>Male = 151	Female = 189<br>Male = 159	2.12 (1)	.15
age 14	14.50 (0.28)	14.48 (0.28)	-0.66 (719.3)	.51
age 17*	17.50 (0.35)	17.49 (0.30)	-0.51 (719.11)	.61
SES	Hard pressed = 33<br>Moderate means = 19<br>Comfortably off = 109<br>Urban prosperity = 22<br>Wealthy achievers = 194	Hard pressed = 18<br>Moderate means = 6<br>Comfortably off = 72<br>Urban prosperity = 30<br>Wealthy achievers = 222	4.04	<.001
prior psychiatric history at age 14	Yes = 113<br>No = 264	Yes = 48<br>No = 300	26.50 (1)	<.001
prior psychiatric history at age 17*	Yes = 174<br>No = 202	Yes = 81<br>No = 267	40.90 (1)	<.001
	**Female (n = 415)**	**Male (n = 310)**	**χ^2 /z / t**	**p-value**
age 14	14.49 (0.27)	14.49 (0.29)	0.11 (642.26)	.91
age 17*	17.50 (0.32)	17.49 (0.33)	0.32 (660.35)	.75
SES	Hard pressed = 26<br>Moderate means = 14<br>Comfortably off = 106<br>Urban prosperity = 23<br>Wealthy achievers = 246	Hard pressed = 25<br>Moderate means = 11<br>Comfortably off = 75<br>Urban prosperity = 29<br>Wealthy achievers = 170	0.93	.35
prior psychiatric history at age 14	Yes = 107<br>No = 308	Yes = 54<br>No = 256	6.71 (1)	<.01
prior psychiatric history at age 17*	Yes = 165<br>No = 250	Yes = 90<br>No = 219	8.32 (1)	<.01

Note. For age we depict the mean values and the belonging standard deviations in brackets. The Pearson's χ^2 tests were used for binary data and performed with Yate's continuity correction. The z-test was used for the SES variable and was conducted as asymptotic linear-by-linear association test, to account for the ordering in the data. The t-tests were used for continuous data and were conducted as Welsh's two-sample t-tests. SES was calculated based on the ACORN classification system (http://www.caci.co.uk).[52] Prior psychiatric history was measured with the Schedule for Affective Disorders and Schizophrenia for School-Age Children (Present and Lifetime Version)[53] and included learning disabilities, clinical sub-threshold diagnoses and deliberate self-harm at age 14; and clinical sub-threshold diagnoses and deliberate self-harm, but not learning disabilities, at age 17. *Please note, one participant has missing data on this variable which is why the numbers do not add up. Tests were conducted two-sided.

Disentangling the Amount of Variance that RFs and Age-14 Distress Explain in Age-17 Distress

First we performed two baseline models, one only included CA (model B1) and the other one CA and gender (model B2) as predictors for age-17 distress. Then we conducted three main models. In addition to CA and gender, the first model contained the ten RFs (model M1), the second model contained age-14 distress (model M2), and the third model contained both the RFs and age-14 distress (model M3) as predictors for age-17 distress. We conducted the three main models for two reasons. Firstly, when comparing the individual effects of the RFs (M1) with the individual effects of age-14 distress (M2) it is possible to find out whether RFs and age-14 distress are similarly predictive for subsequent age-17 distress. This comparison seemed important, as the predictive value of previous distress on future distress has been investigated frequently, but little is known about the predictive magnitude of the RFs. Secondly, exploring the effects of RFs on age-17 distress over and above the effects of age-14 distress (M3) seemed relevant, as it gives an indication for the magnitude with which RFs explain change in distress between age 14 and 17.

Adding the RFs to CA and gender significantly improved the model and increased the explained variance from 10 to 28% (see Likelihood-Ratio test for M1 in Table 2). Similarly, adding age-14 distress (instead of the RFs) to CA and gender significantly improved the model and increased the explained variance from 10 to 38% (see Likelihood-Ratio test for M2 in Table 2; see Supplement IV for Figures depicting change in distress). Adding age-14 distress to the model with CA, gender and the RFs improved the model significantly and increased the explained variance from 28 to 39% (see Likelihood-Ratio test for M3-D14 in Table 2). Adding the RFs to the model with CA, gender and age-14 distress did not improve the model significantly and increased the explained variance from 38 to 39% (see Likelihood-Ratio test for M3-RFs in Table 2). Hence, the RFs seemed to explain age-17 distress, but not the change in distress from age 14 to age 17. Importantly, there was no multicollinearity between the RFs and age-14 distress (see Supplement V). When computing the analyses separately for the CA+ and the CA- group (CA+: M1 = 29%, M2 = 40%, M3 = 42%; CA-: M1 = 25%, M2 = 31%, M3 = 33%), or for females and males (females: M1 = 22%, M2 = 34%, M3 = 35%; males: M1 = 27%, M2 = 33%, M3 = 37%), the result patterns remained similar.

Table 2

Linear regression models

	Model	b	p-value	R^2	R^2 adj	LRT(df)	p-value
Baseline model I (B1): With CA as regressor							
B1	CA	0.39	<.001*	4%	3%		
Baseline model II (B2): Adding gender to B1							
						compared against B1	
B2	gender	-0.52	<.001*	10%	9%	48.14(1)	<.001*
Adding RFs and age-14 distress (D14) separately to B2							
						compared against B2	
M1	RFs	-0.69	-	28%	27%	145.88(10)	<.001*
						compared against B2	
M2	D14	0.61	<.001*	38%	38%	223(1)	<.001*
Adding RFs and age-14 distress (D14) together to B2							
						compared against M2	
M3	RFs	0.11	-	39%	38%	6.93(10)	.43
						compared against M1	

M3	D14	0.64	<.001*	39%	38%	84.05(1)	<.001*

Note. adj = adjusted. LRT = Likelihood-Ratio test. There is no p-value for the RFs in model M1 and M3, as the bs of the RFs are here summed up to illustrate whether the cumulative effect is positive or negative, but as the RFs are 10 individuals regressors there is no cumulative p-value.

Disentangling the Relative Importance of RFs and Age-14 Distress in Explaining Age-17 Distress

We next decomposed the individual variance contribution of the regressors. In the model including both age-14 distress and the RFs, the RFs explained slightly less variance in age-17 distress than age-14 distress (M3 RFs total variance = 42%; M3 age-14 distress total variance = 46%; see Table 3). Moreover, when taking age-14 distress into account the importance ranking of the RFs stayed mainly the same as in the model without age-14 distress (i.e. compare M1 and M3). The self-esteem and brooding RFs explained most and expressive suppression explained the least amount of variance. The results remained similar when being computed separately for CA+ (M3 RFs total variance = 45%; M3 age-14 distress total variance = 48%) and CA- groups (M3 RFs total variance = 47%; M3 age-14 distress total variance = 45%), as well as for female (M3 RFs total variance = 43%; M3 age-14 distress total variance = 52%) and male participants (M3 RFs total variance = 51%; M3 age-14 distress total variance = 46%). The RFs seemed to explain more relative variance in the CA- and the male group, whereas age-14 distress seemed to explain more relative variance in the CA+ and the female group.

Table 3

Relative importance analyses for the whole group: for RFs only (M1), age-14 distress only (M2), and RFs and age-14 distress together (M3)

**Variable**	**% M1: RFs only**	**bootstrap CI**	**% M2: D14 only**	**bootstrap CI**	**% M3: RFs & D14**	**bootstrap CI**
**Abs**	28.03	-	37.79	-	38.66	-
CA	06.93	02.60-12.51	05.88	01.95-12.20	04.40	01.33-09.48
Gender	12.91	05.38-20.26	10.12	05.20-16.56	07.12	03.15-12.00
**Total**	19.83	-	16.00	-	11.51	-
Brooding	23.23	14.52-31.08	-	-	11.36	07.81-15.41
Neg. self-esteem	19.23	11.88-26.11	-	-	10.07	07.65-12.80
Pos. self-esteem	10.23	05.60-16.11	-	-	05.76	03.68-08.63
Family cohesion	08.53	04.50-17.32	-	-	04.52	02.34-07.90
Friendships	06.16	02.26-14.41	-	-	03.10	01.47-06.08
Reflection	05.99	03.37-09.65	-	-	03.43	02.16-05.68
Family support	03.64	01.55-07.47	-	-	02.18	00.96-05.02
Aggression	01.41	00.33-05.22	-	-	00.88	00.54-02.19
Dis. tolerance	01.17	00.70-03.68	-	-	00.84	00.53-02.14
Expressive sup.	00.59	00.09-03.41	-	-	00.22	00.07-02.01
**Total**	80.17	-	-	-	42.36	-
D14	-	-	84.00	76.24-90.26	46.13	37.83-51.53
**Total**	-	-	84.00	-	46.13	-

Note. D14 = age-14 distress, CI = confidence interval, Abs = absolute amount of explained variance, CA = childhood adversity, Neg. = negative, Pos. = positive, Dis. = distress, sup. = suppression.

Disentangling the Accuracy with which RFs and Age-14 Distress Predict Age-17 Distress

We first performed a series of factor mixture models to classify the adolescents based on their categorical distress profiles, while also taking into account the continuous nature of distress. The three-class model, which allows the factor score mean to vary per distress class (called factor mixture model - 1; for more specific analysis details see Supplement II), performed well (entropy = 0.95) and revealed a theoretically plausible solution, splitting the adolescents into "low/mild", "moderate" and "high" distress severity classes. Figure 1 shows the class solution plotted against the continuous general distress scores.

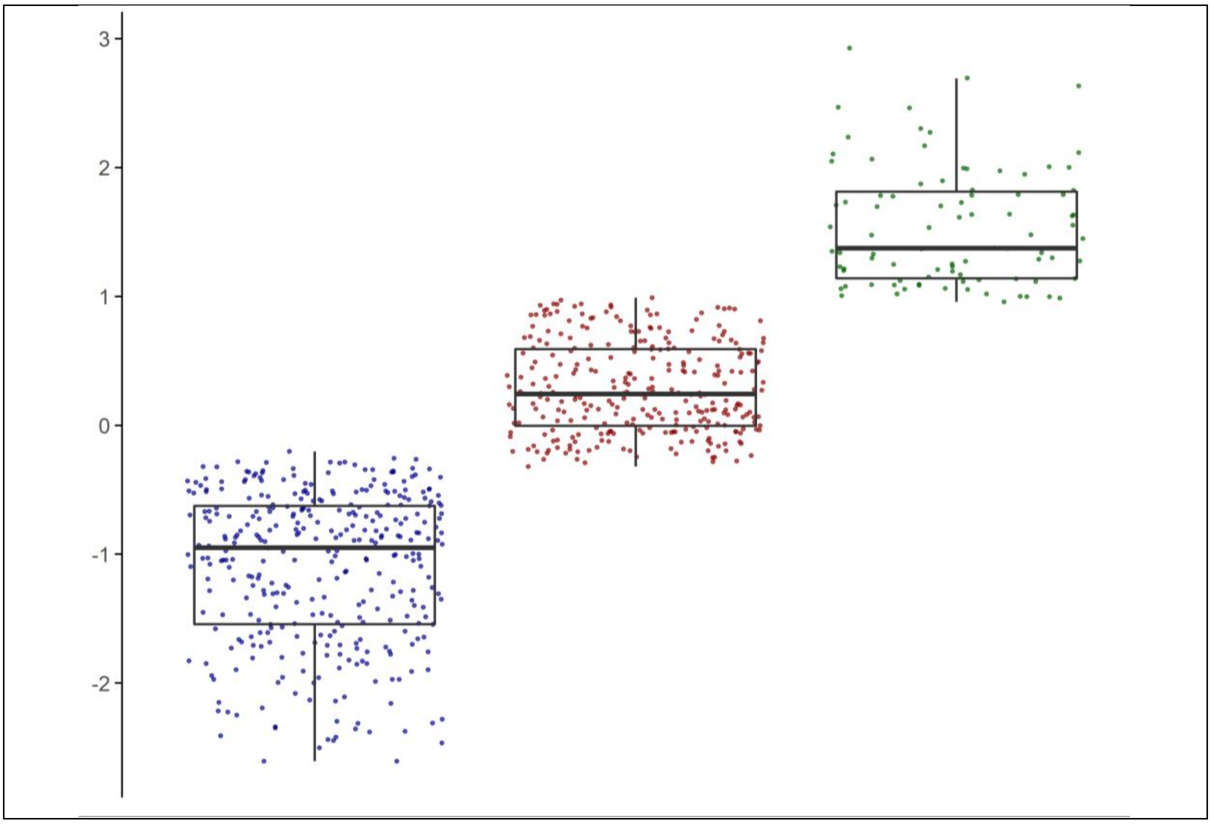

Figure 1. Three-class distress solution (low: n = 343; moderate: n = 292; high: n = 90) plotted against the continuous distress severity scores. Center line = median (50% quantile); lower box limit =25% quantile; upper box limit = 75% quantile; lower whisker = smallest observation greater than or equal to the lower box limit - 1.5 x Inter Quartile Range (IQR); upper whisker = largest observation less than or equal to the upper box limit + 1.5 x Inter Quartile Range (IQR).

As the best class solution was ordered categorical, we conducted three *ordinal* prediction models with the three-class distress variable as outcome variable. Of the three models one again contained the RFs (M1), one age-14 distress (M2) and one both (RFs and age-14 distress; M3) in addition to gender and CA as predictors. Here, we conducted the three models to investigate whether RFs (M1) have a similar predictive accuracy as age-14 distress (M2), and to find out whether the combination of RFs and age-14 distress is better than one information source alone (M3 vs M1 and M2). The applied ordinal regression models have a proportional odds assumption, which was not met for all predictors. Therefore, we conducted the ordinal regressions as partial proportional odds models and relaxed the proportional odds

assumption for those predictors that did not meet the assumption (see details in Supplement VI).

The three models (M1-M3) had a low to acceptable accuracy ranging from 63% to 66% (see Table 4). Hence, about 2 out of 3 adolescents were correctly predicted into their distress severity class, regardless of using RFs, age-14 distress, or both as predictors for age-17 distress. The results were somewhat different when we split the adolescents into CA+ (accuracy: M1 = 45%, M2 = 55%, M3 = 52%), CA- (accuracy: M1 = 67%, M2 = 60%, M3 = 59%), female (accuracy: M1 = 50%, M2 = 56%, M3 = 53%) and male groups (accuracy: M1 = 63%, M2 = 62%, M3 = 65%). Yet, most of the prediction accuracies did formally not differ between the CA and gender subgroups (for details see Supplement VII); only model M1 revealed a significant accuracy difference between the CA subgroups (Chi^2 = 8.16, df = 1, p = 0.004).

Table 4

Ordinal prediction analyses for the whole group: for RFs only (M1), age-14 distress only (M2), and RFs and age-14 distress together (M3)

	**M1: RFs only**		**M2: D14 only**		**M3: RFs & D14**	
	**observed**	**predicted**	**observed**	**predicted**	**observed**	**predicted**
Residual deviance	936.49	-	899.40	-	876.70	-
ROC	-	low=0.77<br>mod=0.68<br>high=0.78	-	low=0.82<br>mod=0.71<br>high=0.82	-	low=0.82<br>mod=0.69<br>high=0.83
Sensitivity	-	low=0.77<br>mod=0.63<br>high=0.09	-	low=0.79<br>mod=0.62<br>high=0.27	-	low=0.80<br>mod=0.60<br>high=0.27
Specificity	-	low=0.70<br>mod=0.66<br>high=0.99	-	low=0.72<br>mod=0.71<br>high=0.98	-	low=0.70<br>mod=0.71<br>high=0.99
Accuracy	-	0.63<br>low=0.73<br>mod=0.65<br>high=0.54	-	0.66<br>low=0.75<br>mod=0.66<br>high=0.62	-	0.66<br>low=0.75<br>mod=0.66<br>high=0.63
Low distress severity	85	94 of which<br>- 65 correct<br>- 26 false mod<br>- 03 false high	85	94 of which<br>- 67 correct<br>- 24 false mod<br>- 03 false high	85	97 of which<br>- 68 correct<br>- 27 false mod<br>- 02 false high
Mod distress severity	73	82 of which<br>- 46 correct<br>- 19 false low<br>- 17 false high	73	76 of which<br>- 45 correct<br>- 18 false low<br>- 13 false high	73	75 of which<br>- 44 correct<br>- 17 false low<br>- 14 false high
High distress severity	22	4 of which<br>- 02 correct<br>- 01 false low<br>- 01 false mod	22	10 of which<br>- 06 correct<br>- 00 false low<br>- 04 false mod	22	8 of which<br>- 06 correct<br>- 00 false low<br>- 02 false mod

Note. D14 = age-14 distress. All models were computed with childhood adversity and gender as predictors. ROC = receiver operating characteristic. Accuracy = relative number of correctly predicted cases. Sensitivity = e.g. for low distress: the number of adolescents who are correctly predicted into the low distress group divided by all adolescent who are actually in the low distress group. Specificity = e.g. for low distress: the number of adolescents who are correctly not predicted into the low distress group divided by all adolescent who are actually not in the low distress group. Variable for which the proportional odds assumption was relaxed can be found in Supplement VI.

We next tested the prediction accuracy for *linear* models with the continuous distress severity variable as outcome measure. These analyses revealed that in contrast to the *ordinal* models, the prediction accuracy for all three *linear* models was low (39 to 47%; Table 5), as the age-17 distress level of only about two in five adolescents was predicted accurately. Similar findings were revealed when splitting the group based on CA (CA+: M1 = 43.01%, M2 = 36.56%, M3 = 43.01%; CA-: M1 = 41.18%, M2 = 50.59%, M3 = 43.53%) and gender (female: M1 = 33.98%, M2 = 36.89%, M3 = 38.84%; male: M1 = 43.42%, M2 = 43.42%, M3 = 46.05%). Once more, the prediction accuracy did not differ significantly between the CA and gender subgroups (see Supplement VII).

Table 5

Linear prediction analyses for the whole group: for RFs only (M1), age-14 distress only (M2), and RFs and age-14 distress together (M3)

	**M1: RFs only**		**M2: D14 only**		**M3: RFs & D14**	
	**observed**	**predicted**	**observed**	**predicted**	**observed**	**predicted**
RMSE	0.92	0.90	0.83	0.83	0.85	0.83
R^2	0.25	0.25	0.38	0.36	0.36	0.35
MAE	0.72	0.71	0.66	0.65	0.67	0.65
Accuracy	-	38.89%	-	42.78%	-	46.67%
predicted into $CI_{95\%}$	-	70	-	77	-	84
not predicted into $CI_{95\%}$	-	110	-	103	-	96
Accuracy plots<br>x-axis:<br>observed (left = black) vs predicted (right = grey) distribution<br>y-axis:<br>distress level	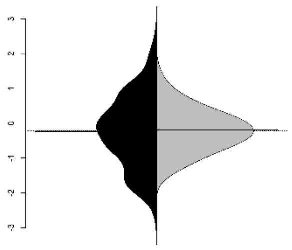		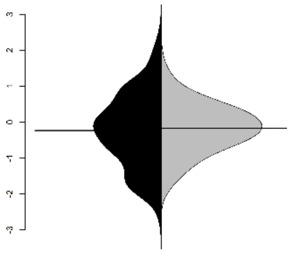		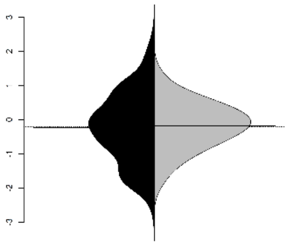	

Note. D14 = age-14 distress. All models were computed with childhood adversity and gender as predictors. RMSE = root mean squared error, MAE = mean absolute error, Accuracy = relative number of correctly predicted cases. Model accuracy was based on 1000 bootstraps.

Post-hoc Exploration: Disentangling the Accuracy for fewer RFs Predicting Age-17 Distress

In our RF regression models (i.e. the M1s), two RFs were (at least marginally < 0.10) significant in three of the four subgroups, namely negative self-esteem and brooding. Moreover those two RFs had in all four subgroups the highest relative importance. Therefore, we next re-ran all prediction models this time instead of including all 10 RFs, CA and gender, only including these two RFs and gender. We did this to investigate whether the assessment of just two RFs and gender would provide similar information as all 10 RFs, CA and gender (i.e. M1). This is important as such an assessment may be more feasible and efficient in many non-clinical settings (e.g. in school assessments). Interestingly, in these post-hoc analyses, both the ordinal and the linear models performed similar as the models including all RFs (change in accuracy: ordinal models from 63% to 61%, $Chi^2 = 0.047$, df = 1, $p = 0.83$; linear models from 38.89%

to 37.78%, $Chi^2 = 0.012$, df = 1, $p = 0.91$). Moreover, the models including gender, the two RFs and age-14 distress were rather comparable to the models including gender, CA, all 10 RFs, and age-14 distress (i.e. M3; change in accuracy: ordinal models from 66% to 66%, $Chi^2 = 0$, df = 1, $p = 1$; linear models from 46.67% to 44.44%, $Chi^2 = 0.101$, df = 1, $p = 0.75$). For completeness, we also conducted the prediction analyses with a subset of the RFs separately in the subgroups, which can be found in Supplement VIII.

DISCUSSION

We aimed to shed light onto potentially promising RF targets that reduce subsequent distress, by pursuing three sub-goals: *First,* we intended to find out to which degree RFs can explain subsequent distress. Our results suggest that RFs explained less variance in age-17 distress than age-14 distress could explain, but when the predictors were used together RFs explained a slightly lower but similar amount of variance than age-14 distress. *Second,* we aimed to find out which RFs are the best indicators for subsequent distress. Our results showed that negative self-esteem and brooding RFs explained most variance and revealed significance in the multivariable regression models. *Third,* we intended to explore with what accuracy RFs can predict distress levels three years later. We found that RFs and distress at age 14 were similarly accurate in predicting distress at age 17, with age-14 distress reaching a slightly higher accuracy. The prediction accuracy was low and highly unsatisfactory when we tried to predict continuous distress scores. When we predicted more crude ordinal ("low", "moderate" and "high") distress classes the accuracy was again not good, but acceptable. As such, both RFs and distress at age 14 (as well as their combination) are able to correctly predict the categorical distress class of about 2 in 3 adolescents.

RFs and/or age-14 distress explained more than one-fourth of the overall variance in distress three years later. Importantly, this was after CA and gender were taken into account. Hence, despite the fact that we have used gender, life-history information (i.e. CA), a broad range of distress symptoms and as many as 10 empirically supported RFs, we could not even explain half of the variance in distress three years later. This is alarming and interesting at the same time. Dinga and colleagues[7] put forward the explanation that the way psychopathology is defined may lack important information (i.e. content validity), such as biological components, which may make it so difficult to predict it well. Another explanation could be derived from the time period we have investigated. We assessed the adolescents during early (age 14) and later (age 17) adolescence, which is generally described as a particularly malleable period during which a lot of mental health problems develop.[3] That is, distress predictions over a period during which many mental health problems manifest themselves may be particularly difficult. A third account may come from the instructions that were provided for the assessment of the distress symptoms: "please tick how often you have felt or acted in this way over the past two weeks". The instructions assess distress during the past two weeks, which for some adolescents may have captured state- rather than trait-distress. An outcome construct that at least to some extent captures state characteristics may complicate the prediction even further. In sum, insufficient content validity, a sensitive developmental time period, and state-like characteristics of the distress variable may all help explain why it was so difficult to predict subsequent distress.

While the RFs explained age-17 distress significantly, the RFs did not explain change in distress from age 14 to age 17 significantly. Yet, the importance ranking of the RFs for explaining age-17 distress remained similar when taking age-14 distress into account. Moreover, the RFs and age-14 distress had a similar relative importance. Importantly, there

was no overlap between RFs and distress items content-wise, and no multicollinearity between RFs and distress at age 14. Besides the comparable relative importance, RFs and age-14 distress had a similar accuracy for predicting age-17 distress. This clearly is a notable finding, as RFs could similarly well predict distress over the course of three years, as distress could predict itself over the course of three years. Moreover, a combination of the two information sources (RFs and age-14 distress) did not necessarily seem advantageous above either source alone. Therefore, if our results were to be replicated, we would assume that knowledge on the RFs may, due to its "conceptual commitment to strengths and assets" (see[54], p. 136), be highly interesting for various public health and clinical settings. More specifically, in settings where a strengths-focus would be more feasible than a symptom-focus, RFs could be assessed to screen, monitor and potentially promote mental health.

If we would have to judge which of the RFs may be the most promising for screening, monitoring and potentially promoting mental health, we probably would choose negative self-esteem and brooding RFs, as those two had the strongest relative importance in reducing the risk of subsequent distress and were significant in the multivariable RF models (M1). Importantly, our prediction results remained rather stable when we used only those two instead of all 10 RFs. Moreover, those two RFs together are measured with only 10 items. Hence, assessing brooding and negative self-esteem RFs would not only have a relatively low stigma risk, but would also be highly time and money efficient. The finding that both self-esteem and brooding seem to play such an important role in the development of mental health problems has been noted in previous research and has led to the suggestion to use self-esteem[55] or brooding[56] as time-efficient and less stigma-prone mental health screens. Young and Dietrich (2014)[56] for example employed the same brooding subscale as used in our study (5 items of the RRS)[38] and detected a screening accuracy of 91 percent for concurrent depressive symptoms in young adolescents. Moreover, both self-esteem and brooding have already been found to be successful intervention targets,[57,58] particularly for interventions aimed at reducing internalizing disorders and/or increasing mental well-being. Interventions targeting self-esteem are suggested to be most successful when provided earlier during adolescence, as self-esteem often is more amenable during early than during late adolescence.[55] Moreover, rumination focused cognitive behaviour therapy has been shown to be a promising prevention intervention for adolescents at risk for internalizing mental health problems.[58] Yet, our results require replication in an independent sample and need ideally to be tested in translational studies, before screening and intervention-related recommendations can be made. Moreover, additional replication in other populations would be ideal, to ensure a clear scope for generalization.

It is important to note that our *linear* prediction models, which are derived from the group level, are not good enough to predict individual-level distress scores three years later. Those models translated for only two in five adolescents correctly to the individual level. Our *categorical* prediction models, which are also derived from the group level, did predict individual-level distress severity classes better, but there is still plenty of room for improvement. Those models translated for about two-third of the adolescents correctly to the individual level. Hence, the generalization from group to individual level is limited, particularly when predicting *continuous* transdiagnostic distress severity. Therefore, it is crucial that future research identifies ways to increase the prediction accuracy for subsequent distress severity. In sum, we recommend that future research (a) examines whether our findings replicate, (b) tests additional RFs that were not measured in our adolescent cohort but are empirically found to reduce subsequent distress, (c) identifies ways which further increase the prediction accuracy (e.g. shorter prediction intervals), (d) is conducted at the individual rather than (or in addition to) the group level, and (e) explores in which prevention and intervention settings targeting RFs may be most helpful.

Last but not least, our study is not without limitations. First, ROOTS has a slightly higher than average SES and thus may mainly generalize to more wealthy populations.[30] Second, our analyses were constrained to those people who provided data for both age 14 and 17, which is not ideal as we cannot rule out a possible increase in selection bias. Third, the binary CA variable may not be ideal as it omits the type of the adversity experience, as well as its severity and frequency. Particularly CA severity may be a valuable consideration and addition in future research.[59] However, justification for using CA as a binary indicator stems from research showing that CAs are likely to co-occur and that clustered CA indices have a robust, negative effect on mental health problems.[32,46,59] For future research it would be ideal if adversity would also be assessed, and controlled, for the interim period between the assessment of the RFs and the assessment of subsequent mental distress. Fourth, the RFs were not all assessed with measures developed to particularly reflect the RF construct at hand (e.g. aggression or expressive suppression). Hence, future research should aim to replicate our results with scales particularly developed for the specific RFs, to increase the content validity. Fifth, we only tested 10 RFs, as only those were assessed in our adolescent cohort. However, in the realm of complexity we think that it would be advantageous if future research could assess and test more than 10 empirically-supported RFs. Sixth, our distress index was mainly defined by internalizing (and not externalizing) symptoms and does not contain information on the distress chronicity. Seventh, we built the prediction models on a subset of the ROOTS cohort (n ~545) to predict distress three years later for another ROOTS subset (n ~180). This means that we used data from the same cohort for training and testing our model. However, it may be that adolescents in our cohort are more comparable to each other than to the general population. This would mean that our prediction accuracy would be lower when using our model to predict distress scores for adolescents who did not take part in ROOTS. Therefore, replication of our findings in a different sample is crucial. Eighth, here we mainly focussed on the overall sample and not so much on findings within the subgroups (CA+ vs. CA-, females vs. males). Yet, there were slight differences in the relative importance of the RFs between the subgroups. Future research should more specifically focus on those differences, for example with moderation analyses.

Critics might argue that investigating age-17 distress as both a categorical and a continuous outcome is superfluous. Yet, we believe that there are good reasons from a scientific as well as a clinical point of view that justify the usage of both (categorical and continuous outcomes) in conjunction. From a statistical point of view it may perhaps seem neater to investigate distress continua. But, first of all our distress classes did take the distress continuum into account, and more importantly, as prior research often only looked at categorical outcomes we feel that it is high time to gain information on the comparison of precise continuous versus more crude categorical outcomes. As our findings showed, it seems like we are not good enough yet to predict precise distress continua, but we are getting into an acceptable range for predicting crude distress classes (from either RFs, distress, or their combination). From a translational point of view, one may favour a categorical outcome as this is often used in clinics, such as cut-offs like "low risk", "at risk/sub-threshold", and "diagnosed". Although crude categorical outcomes may be more easily translatable, providing results of both approaches has given rise to the clinically relevant finding that RFs and prior distress may be promising targets for screens aiming at predicting rough distress risk-categories (e.g. “low”, “moderate”, “high”), but not yet for screens aiming at predicting precise distress risk levels.

As pointed out in the introduction, there is a sparse but ongoing discourse about whether resilience and risk factors are opposing sides of the same coin, which cannot fully be done justice within the scope of this manuscript. However, we suggest that future studies could conduct more idiographic rather than group level research, as the “relationship between resilience and risk factors is likely to additionally depend on biological predispositions, type(s)

of adversity experienced, the specific environmental circumstances, and the developmental stage" (see p. 3 in Supplement XVI of Fritz et al, 2019[18]) of the adolescent. Moreover, while this manuscript specifically focusses on using RFs that predict mental health problems (in individuals with and without CA exposure), it would be interesting to see future research taking the same modelling approach but focussing on those factors that predict a resilient functioning outcome. To this end one could for example focus on resilience predictors reviewed by Kalisch and colleagues (2015; including hair cortisol concentration, trait self-enhancement, expression of specific gene networks, and cortisol stress reactivity),[60] or on factors that predict resilient growth trajectories and resilient functioning outcomes as reviewed by Bonanno and colleagues (2011; including perceived control, high positive affectivity, low negative affectivity, trait resilience, low brooding, coping self-efficacy, emotional support, social support, instrumental support, favorable worldviews, and positive emotions),[61] or on factors that relate to resilient functioning specifically following childhood maltreatment, as reviewed in Ioannidis and colleagues (2020; including the social environment as well as biological factors related to the hypothalamic-pituitary-adrenal axis and polygenetics).[62]

Overall, our results showed that the RFs were able to correctly predict the categorical ('low'/'moderate'/'high') distress class of 2 in 3 adolescents three years later. This finding was highly similar when predicting age-17 from age-14 distress. The two RFs that were most promising in predicting and reducing subsequent distress were negative self-esteem and brooding. Hence, those two RFs may potentially be promising targets for risk-detection and interventions, if they hold up in replication and translational research.

REFERENCES

1. World Health Organization. The European Mental Health Action Plan 2013–2020. https://www.euro.who.int/__data/assets/pdf_file/0020/280604/WHO-Europe-Mental-Health-Acion-Plan-2013-2020.pdf. Published 2015. Accessed July 31, 2020.
2. Tice P, Bose J, Hedden SL, Lipari RN, Park-Lee E, Substance Abuse and Mental Health Services Administration. *Key Substance Use and Mental Health Indicators in the United States: Results from the 2017 National Survey on Drug Use and Health (HHS Publication No. SMA 18-5068, NSDUH Series H-53).* Rockville, MD: Center for Behavioral Health Statistics and Quality, Substance Abuse and Mental Health Services Administration; 2018.
3. World Health Organization. Adolescent mental health in the European Region: Factsheet for World Mental Health Day 2018. https://www.euro.who.int/__data/assets/pdf_file/0005/383891/adolescent-mh-fs-eng.pdf. Published 2018. Accessed July 31, 2020.
4. Ries Merikangas K, He J, Burstein M, et al. Lifetime Prevalence of Mental Disorders in US Adolescents: Results from the National Comorbidity Study-Adolescent Supplement (NCS-A). *J Am Acad Child Adolesc Psychiatry*. 2010;49(10):980-989. doi:10.1016/j.jaac.2010.05.017
5. Lawrie SM, Fletcher-Watson S, Whalley HC, McIntosh AM. Predicting major mental illness: ethical and practical considerations. *Br J Psychiatry Open*. 2019;5(e30):1-5. doi:10.1192/bjo.2019.11
6. Shatte ABR, Hutchinson DM, Teague SJ. Machine learning in mental health: A scoping review of methods and applications. *Psychol Med*. 2019;(49):1426-1448. doi:10.1017/S0033291719000151
7. Dinga R, Marquand AF, Veltman DJ, et al. Predicting the naturalistic course of depression from a wide range of clinical, psychological, and biological data: a machine learning approach. *Transl Psychiatry*. 2018;8:241. doi:10.1038/s41398-018-0289-1
8. Lewis SJ, Arseneault L, Caspi A, et al. The epidemiology of trauma and post-traumatic stress disorder in a representative cohort of young people in England and Wales. *The Lancet Psychiatry*. 2019;6(3):247-256. doi:10.1016/S2215-0366(19)30031-8
9. Pintelas EG, Kotsilieris T, Livieris IE, Pintelas P. A review of machine learning prediction methods for anxiety disorders. In: *Proceedings of the 8th International Conference on Software Development and Technologies for Enhancing Accessibility and Development and Technologies for Enhancing Accessibility and Fighting Info-Exclusion*. Greece: DSAI 2018; 2018:1-8. doi:10.1145/3218585.3218587
10. Afifi TO, MacMillan HL. Resilience Following Child Maltreatment: A Review of Protective Factors. *Can J Psychiatry*. 2011;56(5):266-272.
11. Marriott C, Hamilton-Giachritsis C, Harrop C. Factors Promoting Resilience Following Childhood Sexual Abuse: A Structured, Narrative Review of the Literature. *Child Abus Rev*. 2014;23:17-34. doi:10.1002/car.2258
12. Wright BK, Kelsall HL, Sim MR, Clarke DM, Creamer MC. Support Mechanisms and Vulnerabilities in Relation to PTSD in Veterans of the Gulf War, Iraq War, and Afghanistan Deployments: A Systematic Review. *J Trauma Stress*. 2013;26:310-318. doi:10.1002/jts.21809
13. Traub F, Boynton-Jarrett R. Modifiable Resilience Factors to Childhood Adversity for

Clinical Pediatric Practice. *Pediatrics*. 2017;139(5):e20162569. doi:10.1542/peds.2016-2569

14. Braithwaite EC, O'Connor RM, Degli-Esposti M, Luke N, Bowes L. Modifiable predictors of depression following childhood maltreatment: a systematic review and meta-analysis. *Transl Psychiatry*. 2017;7:e1162. doi:10.1038/tp.2017.140
15. Chen H, Huang Y, Zhang N. Joint modeling of a linear mixed effects model for self-esteem from mean ages 13 to 22 and a generalized linear model for anxiety disorder at mean age 33. *J Med Stat Informatics*. 2015;3(1):1-6. doi:10.7243/2053-7662-3-1
16. Meehan AJ, Latham RM, Arseneault L, Stahl D, Fisher HL, Danese A. Developing an individualized risk calculator for psychopathology among young people victimized during childhood: A population-representative cohort study. *J Affect Disord*. 2020;262:90-98. doi:10.1016/j.jad.2019.10.034
17. Fritz J, de Graaff A-M, Caisley H, van Harmelen A-L, Wilkinson PO. A Systematic Review of Amenable Resilience Factors that Moderate and/or Mediate the Relationship between Childhood Adversity and Mental Health in Young People. *Front Psychiatry*. 2018;9:230. doi:10.3389/fpsyt.2018.00230
18. Fritz J, Stochl J, Fried EI, et al. Unravelling the complex nature of resilience factors and their changes between early and later adolescence. *BMC Med*. 2019;17:203. doi:10.1186/s12916-019-1430-6
19. van Harmelen A-L, Gibson JL, St Clair MC, et al. Friendships and Family Support Reduce Subsequent Depressive Symptoms in At-Risk Adolescents. *PLoS One*. 2016;11(5):e0153715. doi:10.1371/journal.pone.0153715
20. Harding KA, Mezulis A. Is Rumination a Risk and a Protective Factor? *Eur J Psychol*. 2017;13(1):28-46. doi:10.5964/ejop.v13i1.1279
21. Diehl M, Hay EL, Chui H. Personal Risk and Resilience Factors in the Context of Daily Stress. *Annu Rev Gerontol Geriatr*. 2012;32(1):251-274. doi:10.1891/0198-8794.32.251
22. Scheffer M, Bolhuis JE, Borsboom D, et al. Quantifying resilience of humans and other animals. *PNAS*. 2018;115(47):11883-11890. doi:10.1073/pnas.1810630115
23. Fritz J, Fried EI, Goodyer IM, Wilkinson PO, van Harmelen A-L. A Network Model of Resilience Factors for Adolescents with and without Exposure to Childhood Adversity. *Sci Rep*. 2018;8:15774. doi:10.1038/s41598-018-34130-2
24. Caspi A, Houts RM, Belsky DW, et al. The p Factor: One General Psychopathology Factor in the Structure of Psychiatric Disorders? *Clin Psychol Sci*. 2014;2(2):119-137. doi:10.1177/2167702613497473
25. Ronald A. The psychopathology p factor: will it revolutionise the science and practice of child and adolescent psychiatry? *J Child Psychol Psychiatry*. 2019;60(5):497-499. doi:10.1111/jcpp.13063
26. St Clair MC, Neufeld S, Jones PB, et al. Characterising the latent structure and organisation of self-reported thoughts, feelings and behaviours in adolescents and young adults. *PLoS One*. 2017;12(4):e0175381. doi:10.1371/journal.pone.0175381
27. Stochl J, Khandaker GM, Lewis G, et al. Mood, anxiety and psychotic phenomena measure a common psychopathological factor. *Psychol Med*. 2015;45:1483-1493. doi:10.1017/S003329171400261X
28. Brodbeck J, Abbott RA, Goodyer IM, Croudace TJ. General and specific components

of depression and anxiety in an adolescent population. *BMC Psychiatry*. 2011;11:191. doi:10.1186/1471-244X-11-191

29. Clark SL, Muthén B, Kaprio J, D'Onofrio BM, Viken R, Rose RJ. Models and Strategies for Factor Mixture Analysis: An Example Concerning the Structure Underlying Psychological Disorders. *Struct Equ Model A Multidiscip J*. 2013;20(4):681-703. doi:10.1080/10705511.2013.824786

30. Goodyer IM, Croudace T, Dunn V, Herbert J, Jones PB. Cohort Profile: Risk patterns and processes for psychopathology emerging during adolescence: the ROOTS project. *Int J Epidemiol*. 2010;39:361-369. doi:10.1093/ije/dyp173

31. McLaughlin KA. Future Directions in Childhood Adversity and Youth Psychopathology. *J Clin Child Adolesc Psychol*. 2016;45(3):361-382. doi:10.1080/15374416.2015.1110823

32. Kessler RC, McLaughlin KA, Greif Green J, et al. Childhood adversities and adult psychopathology in the WHO World Mental Health Surveys. *Br J Psychiatry*. 2010;197:378-385. doi:10.1192/bjp.bp.110.080499

33. McLaughlin KA, Greif Green J, Gruber MJ, Sampson NA, Zaslavsky AM, Kessler RC. Childhood Adversities and First Onset of Psychiatric Disorders in a National Sample of US Adolescents. *Arch Gen Psychiatry*. 2012;69(11):1151-1160. doi:10.1001/archgenpsychiatry.2011.2277

34. Greif Green J, McLaughlin KA, Berglund PA, et al. Childhood adversities and adult psychopathology in the National Comorbidity Survey Replication (NCS-R) I: Associations with first onset of DSM-IV disorders. *Arch Gen Psychiatry*. 2010;67(2):113. doi:10.1001/archgenpsychiatry.2009.186.

35. Goodyer IM, Wright C, Altham PME. Recent friendships in anxious and depressed school age children. *Psychol Med*. 1989;19(1):165-174. doi:10.1017/S0033291700011119

36. Epstein NB, Baldwin LM, Bishop DS. The McMaster Family Assessment Device. *J Marital Fam Ther*. 1983;9(2):171-180.

37. Rosenberg M. *Society and the Adolescent Self-Image*. Princeton, NJ: Princeton University Press; 1965.

38. Treynor W, Gonzalez R, Nolen-Hoeksema S. Rumination reconsidered: A psychometric analysis. *Cognit Ther Res*. 2003;27(3):247-259.

39. Burwell RA, Shirk SR. Subtypes of rumination in adolescence: Associations between brooding, reflection, depressive symptoms, and coping. *J Clin Child Adolesc Psychol*. 2007;36(1):56-65. doi:10.1080/15374410709336568

40. Goodyer IM, Tsancheva S, Byford S, et al. Improving mood with psychoanalytic and cognitive therapies (IMPACT): a pragmatic effectiveness superiority trial to investigate whether specialised psychological treatment reduces the risk for relapse in adolescents with moderate to severe unipolar dep... *Trials*. 2011;12:175. doi:10.1186/1745-6215-12-175

41. American Psychiatric Association. *Diagnostic and Statistical Manual of Mental Disorders (4th Ed., Text Rev.)*. Washington, DC: Author; 2000.

42. Bould H, Joinson C, Sterne J, Araya R. The Emotionality Activity Sociability Temperament Survey: Factor analysis and temporal stability in a longitudinal cohort. *Pers Individ Dif*. 2013;54:628-633. doi:10.1016/j.paid.2012.11.010

43. Poythress NG, Douglas KS, Falkenbach D, et al. Internal Consistency Reliability of the Self-Report Antisocial Process Screening Device. *Assessment*. 2006;13(1):107-113. doi:10.1177/1073191105284279

44. Reynolds CR, Richmond BO. What I Think and Feel: A Revised Measure of Children's Manifest Anxiety. *J Abnorm Child Psychol*. 1978;6(2):271-280.

45. Messer SC, Angold A, Costello EJ. Development of a Short Questionnaire for Use in Epidemiological Studies of Depression in Children and Adolescents: Factor Composition and Structure across Development. *Int J Methods Psychiatr Res*. 1995;5:251-262.

46. Dunn VJ, Abbott RA, Croudace TJ, et al. Profiles of family-focused adverse experiences through childhood and early adolescence: The ROOTS project a community investigation of adolescent mental health. *BMC Psychiatry*. 2011;11:109. doi:10.1186/1471-244X-11-109

47. Wu H, Estabrook R. Identification of Confirmatory Factor Analysis Models of Different Levels of Invariance for Ordered Categorical Outcomes. *Psychometrika*. 2016;81(4):1014-1045. doi:10.1007/s11336-016-9506-0

48. Groemping U. Relative Importance for Linear Regression in R: The Package relaimpo. *J Stat Softw*. 2006;17(1):1-27. http://www.jstatsoft.org/.

49. Lindeman RH, Merenda PF, Gold RZ. *Introduction to Bivariate and Multivariate Analysis.* Glenview, IL: Scott, Foresman and Company; 1980.

50. R Core Team. R: A language and environment for statistical computing. *R Found Stat Comput Vienna,*. 2018:Austria. Retrieved from https://www.R-project.org/.

51. Muthén LK, Muthén BO. *Mplus User's Guide. Eights Edition.* Los Angeles, CA: Muthén & Muthén; 2017.

52. Morgan M, Chinn S. ACORN group, social class, and child health. *J Epidemiol Community Health*. 1983;37:196-203.

53. Kaufman J, Birmaher B, Brent D, et al. Schedule for Affective Disorders and Schizophrenia for School-Age Children-Present and Lifetime Version (K-SADS-PL): Initial reliability and validity data. *J Am Acad Child Adolesc Psychiatry*. 1997;36(7):980-988. doi:10.1097/00004583-199707000-00021

54. Luthar SS, Lyman EL, Crossman EJ. Resilience and Positive Psychology. In: Lewis M, Rudolph KD, eds. *Handbook of Developmental Psychopathology: Third Edition*. New York: Springer Science+Business Media; 2014:125-140. doi:10.1007/978-1-4614-9608-3_7

55. Keane L, Loades M. Review: Low self-esteem and internalizing disorders in young people – a systematic review. *Child Adolesc Ment Health*. 2017;22(1):4-15. doi:10.1111/camh.12204

56. Young CC, Dietrich MS. Screening for Rumination and Brooding May Be a Feasible Method of Identifying Adolescents at High Risk for Depression. *J Pediatr Nurs*. 2014;29:688-695. doi:10.1016/j.pedn.2014.04.010

57. Millar SL, Donnelly M. Promoting mental wellbeing: developing a theoretically and empirically sound complex intervention. *J Public Health (Bangkok)*. 2013;36(2):275-284. doi:10.1093/pubmed/fdt075

58. Watkins E. Psychological treatment of depressive rumination. *Curr Opin Psychol*. 2015;4:32-36. doi:10.1016/j.copsyc.2015.01.020

59. Schlechter P, Fritz J, Wilkinson PO. The Youth and Childhood Adversity Scale: A Comparison of Presence vs. Absence, Severity and Frequency Indicators. *Open Sci Framew Prepr*. 2019. https://doi.org/10.31219/osf.io/zf8r9.

60. Kalisch R, Müller MB, Tüscher O. A conceptual framework for the neurobiological study of resilience. *Behav Brain Sci*. 2015;38:e92. doi:10.1017/S0140525X1400082X

61. Bonanno GA, Westphal M, Mancini AD. Resilience to Loss and Potential Trauma. *Annu Rev Clin Psychol*. 2011;7:511-535. doi:10.1146/annurev-clinpsy-032210-104526

62. Ioannidis K, Askelund AD, Kievit R, van Harmelen A-L. The complex neurobiology of resilient functioning after childhood maltreatment. *BMC Med*. 2020;(18:32). doi:10.1186/s12916-020-1490-7

63. Kampstra P. Beanplot: A Boxplot Alternative for Visual Comparison of Distributions. *J Stat Softw*. 2008;28(1):1-9. http://www.jstatsoft.org/v28/c01/.

64. Schlegel B, Steenbergen M. brant: Test for Parallel Regression Assumption. R package version 0.2-0. 2018. https://cran.r-project.org/package=brant.

65. Fox J, Weisberg S. *An {R} Companion to Applied Regression (Second Edition).* CA: Thousand Oaks: Sage.; 2011.

66. Kuhn M. caret: Classification and Regression Training. 2018. https://cran.r-project.org/package=caret/.

67. Hothorn T, Hornik K, van de Wiel MA, Zeileis A. Implementing a Class of Permutation Tests: The coin Package. *J Stat Softw*. 2008;28(8):1-23. http://www.jstatsoft.org/v28/i08/.

68. Wickham H, François R, Henry L, Müller K. dplyr: A Grammar of Data Manipulation. R package version 0.7.7. https://cran.r-project.org/package=dplyr. Published 2018.

69. R Core Team. foreign: Read Data Stored by “Minitab”, “S”, “SAS”, “SPSS”, “Stata”, “Systat”, “Weka”, “dBase”, ... R package version 0.8-70. 2017. https://cran.r-project.org/package=foreign.

70. Harrell Jr. FE. Hmisc: Harrell Miscellaneous. 2018. https://cran.r-project.org/package=Hmisc.

71. Venables WN, Ripley BD. *Modern Applied Statistics with S (Fourth Edition)*. New York: Springer; 2002.

72. Yan Y. MLmetrics: Machine Learning Evaluation Metrics. 2016. https://cran.r-project.org/package=MLmetrics.

73. Grosjean P, Ibanez F. pastecs: Package for Analysis of Space-Time Ecological Series. R package version 1.3.21. https://cran.r-project.org/package=pastecs. Published 2018.

74. Robin X, Turck N, Hainard A, et al. pROC: An open-source package for R and S+ to analyze and compare ROC curves. *BMC Bioinformatics*. 2011;12:77. doi:10.1186/1471-2105-12-77

75. Epskamp S, Cramer AOJ, Waldorp LJ, Schmittmann VD, Borsboom D. qgraph: Network Visualizations of Relationships in Psychometric Data. *J Stat Softw*. 2012;48(4):1-18. http://www.jstatsoft.org/.

76. Wickham H. Reshaping data with the reshape package. *J Stat Softw*. 2007;21(12):2007.

77. Jorgensen TD, Pornprasertmanit S, Schoemann AM, Rosseel Y. semTools: Useful tools for structural equation modeling. R package version 0.5-1.933. https://cran.r-

project.org/package=semTools. Published 2018.

78. Yee TW. The VGAM Package for Categorical Data Analysis. *J Stat Softw*. 2010;32(10):1-34. http://www.jstatsoft.org/v32/i10/.

Acknowledgements

We are extremely grateful (a) for advice regarding the imputation analyses (directly or indirectly for related projects) from Kyle Lang, Todd Little, Luke Waggenspack, Whitney Moore, Rogier Kievit, and Terrence Jorgensen, as well as (b) for being pointed toward relative importance analyses by Eiko Fried.

Funding

JS received support from the NIHR Collaboration for Leadership in Applied Health Research and Care (CLAHRC) East of England (EoE) at the Cambridgeshire and Peterborough NHS Foundation Trust. IMG is funded by a Wellcome Trust Strategic Award and declares consulting to Lundbeck. ALvH is funded by the Royal Society (DH15017 & RGF\EA\180029 & RFG/RI/180064), and MQ (MQBFC/2). JF is funded by the Medical Research Council Doctoral Training/Sackler Fund and the Pinsent Darwin Fund. The views expressed are those of the authors and not necessarily those of the NHS, the NIHR, or the Department of Health and Social Care. Funders of the authors played no role in the study conduction, analysis performance, or the reporting of the study.

Author Contributions

IMG was responsible for the data collection. JF designed and formulated the research proposal (i.e. research questions and analysis plan), performed the analyses, and conducted the writing. JS, ALvH, and POW commented on the research proposal and the manuscript. All authors approved the final manuscript. ALvH and POW are joint last authors of this manuscript.

Competing Interests

All authors declare that they have no competing interests.

SUPPLEMENTS

Supplement I

(Longitudinal) Confirmatory Factor Analyses Conducted with WLSMV estimator

Model	Robust CFI	Robust TLI	Robust RMSEA	RMSEA 90% CI	Chisq(df)
Friendship support [35]***, 5 items, 1 unique item covariance, n = 1138***					
**BM**	0.994	0.985	0.073	0.049– 0.100	14.44(4)
Family support [36]***, 5 items, 1 unique item covariance, n = 1122***					
**BM**	0.995	0.987	0.078	0.054-0.105	10.87(4)
Family cohesion [36]***, 7 items, 1 unique item covariance, n = 1129***					
**BM**	0.982	0.970	0.081	0.067-0.095	53.83(13)
Positive self-esteem [37]***, 5 items, 1 unique item covariance, n = 1148***					
**BM**	0.998	0.994	0.083	0.059-0.109	09.99(4)
Negative self-esteem [37]***, 5 items, 0 unique item covariances, n = 1151***					
**BM**	0.999	0.997	0.040	0.016-0.065	05.61(5)
Brooding [38]***, 5 items, 0 unique item covariances, n = 1145***					
**BM**	0.994	0.988	0.068	0.046-0.091	13.79(5)
Reflection [38]***, 5 items, 1 unique item covariance, n = 1148***					
**BM**	0.999	0.999	0.024	0.000-0.055	3.455(4)
Distress tolerance [42]***, 5 items, 1 unique item covariance, n = 1149***					
**BM**	0.982	0.956	0.140	0.117-0.166	39.928(4)
Aggression [40]***, 4 items, 0 unique item covariances, n = 1156***					
**BM**	0.999	0.998	0.015	0.000-0.062	00.887(2)
Distress [44,45]***, 41 items, 2 unique item covariances, n = 865***					
**C IM**	0.966	0.964	0.027	0.025-0.028	5167.43(3193)
**L+T+I IM**	0.964	0.964	0.027	0.025-0.028	5357.30(3314)

Note. WLSMV = weighted least squares mean and variance corrected estimator; CFI = Comparative fit index; TLI = Tucker-Lewis index; RMSEA = Root mean square error of approximation; CI = Confidence interval; chisq = chi-square; BM = baseline model; C IM = configural invariance model; L+T+I IM = loadings, thresholds, and intercepts invariance model. All models were conducted with the delta parameterization.

We used modification indices only when statistically necessary and theoretically defensible. All CFA models fitted reasonably. For aggression the resulting factor scores were notably poorly distributed and we therefore binarized this variable. The continuous latent distress scores used in the manuscript are based on a strongly invariant, categorical CFAs (i.e. L+T+I IM models in the above table), to ensure the latent mean comparability between distress at age 14 and age 17. More specifically, we applied the delta parametrization, equated item loadings and item thresholds across the two time points (i.e. age 14 and 17), fixed all item intercepts to 0, the item scales of the first time point to 1, the latent factor mean of the first time point to 0, and the latent factor variance of the first time point to 1.

Supplement II

For the categorical prediction model we aimed to classify the adolescents based on their distress profiles into a categorical distress variable. Firstly, we applied latent class analysis (LCA) with ordinal items, an MLR estimator, and a logit link (see Table 1), to identify possible class solutions. We used the same 41 anxiety and depression items for the LCA as for the general distress factor model. We tested a 2-, a 3- and a 4-class solution. The 3-class solution had the highest entropy (=0.961), but did not differ significantly from the 2-class solution (entropy = 0.960; Likelihood-Ratio tests (LRT) = 2690.16, $p = 0.76$). Based on those results we conducted a series of factor mixture models (FMMs),[29] which are hybrid models that add latent classes on top of the latent factors, with different invariance levels between the classes. We tested those FMMs with 2, 3 and 4 classes. The FMM1 is the factor mixture model with the most invariance constraints between classes, as it only allows the factor mean to vary between classes. The FMM1 with 2 classes fitted better than the FMM1 with 1 class (LRT = 7462.22, $p < .001$).

Moreover, the FMM1 with 3 classes fitted better than the FMM1 with 2 classes (LRT = 2143.07, $p < .01$). The FMM1 with 4 classes fitted better than the FMM1 with 3 classes (LRT = 906.57, $p < .05$), but had a lower entropy (0.952 vs 0.922) and revealed one very small class (class 4). In the prediction models, 32 adolescents of this class were sampled in the training and 10 in the test sample. Hence, this is already a small group to be predicted, but when we then split the sample further into CA+ vs CA- and into female vs male, the high distress class had for the CA- group only 6 adolescents in the training and 2 in the test sample. Similarly, the female group had only 5 adolescents in the training and 1 in the test sample. We therefore considered this class practically too small. We also tested the FMM2 model, in which in addition to the factor mean also the factor variance can vary between classes. The FMM2 model for the 2, the 3 and the 4 class solution had however a noticeably low entropy (2 classes: 0.371; 3 classes: 0.532, 4 classes = 0.314) and did not fit better than a 1 class solution. In sum, we decided to go forward with the FMM1 3-class solution, to have sufficiently predictable class sizes. Moreover, the 3 class model revealed a theoretically plausible and practical solution, which is described in the main text. For completeness we also computed the prediction analyses with the FMM1 with 4 classes as outcome variable, which can be found in Supplement IX.

Latent Class Analyses with MLR estimator and logit link

classes	AIC	BIC	BIC_{adj}	Entropy	LMR LRT	p-value	Class counts
2	49999.07	51175.47	50391.06	0.960	08773.84	.415	1=567; 2=298
3	47553.71	49320.68	48142.48	0.961	02690.16	.764	1=115; 2=408; 3=342
4	46660.62	49018.18	47446.18	0.937	01139.73	.783	1=58; 2=187; 3=300, 4=320

Note. AIC =Akaike information criterion. BIC =Bayesian information criterion. BIC_{adj} = sample size adjusted BIC. LMR LRT = Lo-Mendel-Rubin adjusted likelihood ratio test for class comparisons.

One-Factor Mixture Models with MLR estimator and logit link

classes	AIC	BIC	BIC_{adj}	Entropy	LMR LRT	p-value	Class counts
FMM1: loadings = class invariant; thresholds = class invariant; factor mean = varying per class (fixed to 0 in 1 class for identification); factor variance = fixed to 0							
2	50053.81	50839.66	50315.66	0.955	07462.22	<0.001	1=304; 2=561
3	47597.85	48393.23	47862.88	0.952	02143.07	<0.01	1=349; 2=405; 3=111
4	46561.22	47366.12	46829.42	0.922	00906.57	< 0.05	1=194; 2=312; 3=307; 4=52
FMM2: loadings = class invariant; thresholds = class invariant; factor mean = varying per class (fixed to 0 in 1 class for identification); factor variance = varying per class							
2	45835.90	46631.27	46100.92	0.371	00006.48	.272	1=0; 2=865
3	45840.23	46649.90	46110.02	0.532	00002.85	.573	1=173; 2=0; 3=692
4	45831.73	46655.68	46106.27	0.314	00016.78	.368	1=499; 2=267; 3=71; 4=28

Note. AIC =Akaike information criterion. BIC =Bayesian information criterion. BIC_{adj} = sample size adjusted BIC. LMR LRT = Lo-Mendel-Rubin adjusted likelihood ratio test for class comparisons. [NI] = the model was not identified.

Supplement III

**Package (version number)**	**Reference**
beanplot (1.2)	Peter Kampstra (2008). Beanplot: A Boxplot Alternative for Visual Comparison of Distributions. Journal of Statistical Software, Code Snippets 28(1). 1-9. http://www.jstatsoft.org/v28/c01/.[63]
brant (0.2-0)	Benjamin Schlegel and Marco Steenbergen (2018). brant: Test for Parallel Regression Assumption. R package version 0.2-0. https://CRAN.R-project.org/package=brant[64]
car (3.0-2)	John Fox and Sanford Weisberg (2011). An {R} Companion to Applied Regression, Second Edition.

	Thousand Oaks CA: Sage.[65] http://socserv.socsci.mcmaster.ca/jfox/Books/Companion
caret (6.0-81)	Max Kuhn (2018). caret: Classification and Regression Training. https://CRAN.R-project.org/package=caret[66]
coin (1.2-2)	Torsten Hothorn, Kurt Hornik, Mark A. van de Wiel, Achim Zeileis (2008). Implementing a Class of Permutation Tests: The coin Package. Journal of Statistical Software 28(8), 1-23. http://www.jstatsoft.org/v28/i08/.[67]
dplyr (0.7.7)	Hadley Wickham, Romain François, Lionel Henry and Kirill Müller (2018). dplyr: A Grammar of Data Manipulation. https://CRAN.R-project.org/package=dplyr[68]
foreign (0.8-70)	R Core Team (2017). foreign: Read Data Stored by 'Minitab', 'S', 'SAS', 'SPSS', 'Stata', 'Systat', 'Weka', 'dBase', ... R package version 0.8-70. https://CRAN.R-project.org/package=foreign[69]
Hmisc (4.1-1)	Frank E Harrell Jr, with contributions from Charles Dupont and many others. (2018). Hmisc: Harrell Miscellaneous. https://CRAN.R-project.org/package=Hmisc[70]
MASS (7.3-50)	Venables, W. N. & Ripley, B. D. (2002) Modern Applied Statistics with S. Fourth Edition. Springer, New York. ISBN 0-387-95457-0[71]
MLmetric (1.1.1)	Yachen Yan (2016). MLmetrics: Machine Learning Evaluation Metrics. https://CRAN.R-project.org/package=MLmetrics[72]
pastecs (1.3.21)	Philippe Grosjean and Frederic Ibanez (2018). pastecs: Package for Analysis of Space-Time Ecological Series. https://CRAN.R-project.org/package=pastecs[73]
pROC (1.14.0)	Xavier Robin, Natacha Turck, Alexandre Hainard, Natalia Tiberti, Frédérique Lisacek, Jean-Charles Sanchez and Markus Müller (2011). pROC: an open-source package for R and S+ to analyze and compare ROC curves. BMC Bioinformatics, 12, p. 77. doi:10.1186/1471-2105-12-77[74]
qgraph (1.5)	Sacha Epskamp, Angelique O. J. Cramer, Lourens J. Waldorp, Verena D. Schmittmann, Denny Borsboom (2012). qgraph: Network Visualizations of Relationships in Psychometric Data. Journal of Statistical Software, 48(4), 1-18. http://www.jstatsoft.org/v48/i04/.[75]
relaimpo (2.2-3)	Ulrike Groemping (2006). Relative Importance for Linear Regression in R: The Package relaimpo. Journal of Statistical Software, 17(1), 1-27.[48]
reshape (0.8.8)	H. Wickham. Reshaping data with the reshape package. Journal of Statistical Software, 21(12), 2007.[76]
semTools (0.5-1.933)	Jorgensen, T. D., Pornprasertmanit, S., Schoemann, A. M., & Rosseel, Y. (2019). semTools: Useful tools for structural equation modeling. R package version 0.5-

	1.933. Retrieved from https://CRAN.R-project.org/package=semTools[77]
VGAM (1.1-1)	Thomas W. Yee (2010). The VGAM Package for Categorical Data Analysis. Journal of Statistical Software, 32(10), 1-34. http://www.jstatsoft.org/v32/i10/.[78]

Supplement IV

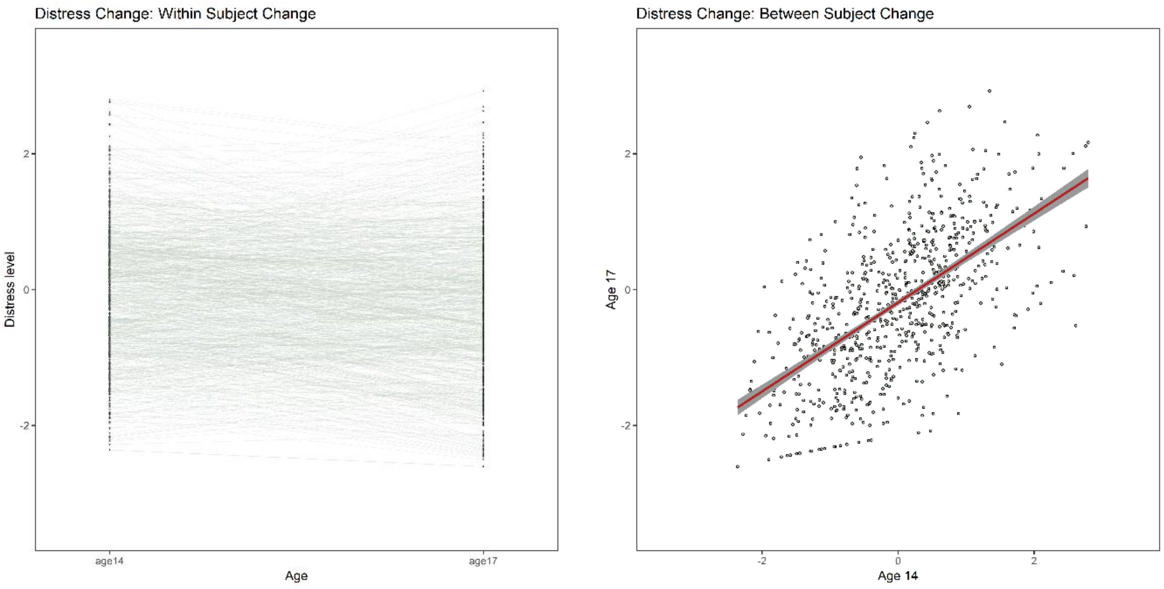

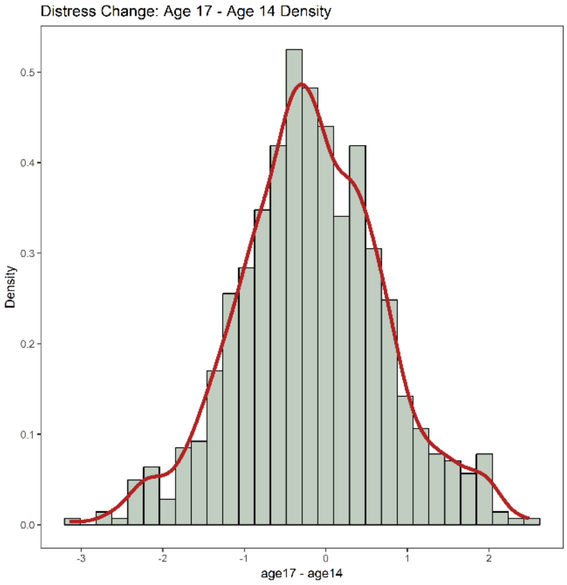

The upper left panel shows the within-subject (idiographic) change in distress from age 14 to age 17. Many adolescents have a rather stable trajectory, but others increase or decrease in distress. This largely variable pattern would also be expected in a naturalistic population sample. The upper right panel depicts the between-subject (nomothetic) association between age-14 and age-17 distress. The positive association indicates that on average adolescents with high age-14 distress also have high age-17 distress. The lower left panel shows that change in distress between age 14 and 17 (i.e. age-17 minus age-14 scores) is overall normally distributed. The plots indicate that there is variability in the change of distress that could be explained by resilience factors.

Supplement V

Variance inflation factors

Mod	CA	gender	Frn	Fms	Fmc	Ngt	Pst	Brd	Rfl	Dst	Agg	Exp	D14
B2	1.00	1.00											

M1	1.09	1.20	1.17	1.88	2.06	2.20	1.83	2.13	1.66	1.13	1.12	1.07	
M2	1.02	1.07											1.08
M3	1.09	1.23	1.21	1.88	2.12	2.63	1.87	2.37	1.67	1.13	1.15	1.08	3.14

Note. Mod = model; CA = childhood adversity; Frn = friendship support; Fms = family support; Fmc = family cohesion; Ngt = negative self-esteem; Pst = positive self-esteem; Brd = brooding; Rfl = reflection; Dst = distress tolerance; Agg = aggression; Exp = expressive suppression; D14 = age-14 distress; B2 = baseline model with CA and gender as predictors; M1 = main model with CA, gender and RFs as predictors; M2 = main model with CA, gender and age-14 distress as predictors; M3 = main model with CA, gender, RFs and age-14 distress as predictors. When taking the square root of the variance inflation factors, none is bigger than 2, which additionally underpins the absence of multicollinearity.

Supplement VI

Ordered categorical, or proportional odds models, have a "proportional odds" or also called "parallel slopes" assumption. This assumption necessitates that when the tested ordinal categories are dichotomized (e.g. here "a": low vs moderate and high, and "b": low and moderate vs high) the logistic prediction of the respective dichotomized categories results in two slopes (i.e. one for scenario "a" and one for scenario "b") that do not differ significantly from each other. If the slopes differ significantly, the proportional odds assumption does not hold and needs to be relaxed. The assumption can be determined for each predictor in the model and only for those predictors that do not meet the assumption separate slope values need to be estimated. This then results in a partial proportional odds model. It would also be possible to estimate a non-proportional odds model to circumvent the assumption for every variable in the model. However, this would be highly disadvantageous as it requires a vast amount of power. Hence we opted for the partial proportional odds model to ensure that we have as much power as possible. The below table depicts all the variables for which the proportional odds assumption was relaxed:

	**M1: RFs only**	**M2: D14 only**	**M3: RFs & D14**
**Whole sample**			
3-class models	-expressive suppression	x	-expressive suppression
3-class (models with reduced number of RFs)	x	-	x
4-class model	x	x	-brooding
**CA+ sample**			
3-class models	-expressive suppression	x	x
3-class (models with reduced number of RFs)	x	-	x
4-class model	x	x	-reflection
**CA- sample**			
3-class models	-reflection	x	-reflection
3-class (models with reduced number of RFs)	x	-	x
4-class model	-positive self-esteem<br>-brooding	x	-positive self-esteem<br>-brooding
**Female sample**			
3-class models	x	x	x
3-class (models with reduced number of RFs)	x	-	x
4-class model	x	x	x
**Male sample**			
3-class models	X	X	-negative self-esteem

3-class (models with reduced number of RFs)	X	-	-negative self-esteem
4-class model	X	X	X

Note. – means not tested. X means that all variables met the proportional odds assumption.

Supplement VII

The below tables depict the prediction accuracy of the prediction models described in the main manuscript. The first two tables depict subgroup accuracy comparisons for CA and gender models, respectively. The third table depicts accuracy comparisons for models including all RFs versus models that only include a subset of the RFs.

Subgroup accuracy comparisons for childhood adversity (CA) models

**Model**	**Coefficient**	**CA+**	**CA-**	**Proportion test summary**
M1 ordinal	Accuracy	45%	67%	Chi-squared = 8.1604, df = 1, p-value = 0.00428
	Correct predictions	42	57	
	Total predictions	94	85	
M2 ordinal	Accuracy	55%	60%	Chi-squared = 0.23164, df = 1, p-value = 0.6303
	Correct predictions	52	51	
	Total predictions	94	85	
M3 ordinal	Accuracy	52%	59%	Chi-squared = 0.56141, df = 1, p-value = 0.454
	Correct predictions	49	50	
	Total predictions	94	85	
M1 linear	Accuracy	43.01%	41.18%	Chi-squared = 0.009141, df = 1, p-value = 0.924
	Correct predictions	40	35	
	Total predictions	93	85	
M2 linear	Accuracy	36.56%	50.59%	Chi-squared = 3.0124, df = 1, p-value = 0.0826
	Correct predictions	34	43	
	Total predictions	93	85	
M3 linear	Accuracy	43.01%	43.53%	Chi-squared = 2.0789e-30, df = 1, p-value = 1
	Correct predictions	40	37	
	Total predictions	93	85	
M1 ordinal 4 classes	Accuracy	46%	52%	Chi-squared = 0.54765, df = 1, p-value = 0.459
	Correct predictions	42	45	
	Total predictions	92	86	
M2 ordinal 4 classes	Accuracy	47%	57%	Chi-squared = 1.4781, df = 1, p-value = 0.2241
	Correct predictions	43	49	
	Total predictions	92	86	
M3 ordinal 4 classes	Accuracy	47%	56%	Chi-squared = 1.1243, df = 1, p-value = 0.289
	Correct predictions	43	48	
	Total predictions	92	86	

Note. M1 = Model 1 contains the ten RFs, M2 = Model 2 contains age-14 distress, M3 = Model 3 contains both the RFs and age-14 distress as predictors for age-17 distress. Correct predictions = number of correctly predicted adolescents, Total predictions = number of adolescents that could have been predicted correctly, Accuracy = ratio correct predictions divided by total predictions. df = degrees of freedom.

Subgroup accuracy comparisons for gender models

**Model**	**Coefficient**	**female**	**male**	**Proportion test summary**
M1 ordinal	Accuracy	50%	63%	Chi-squared = 2.7644, df = 1, p-value = 0.09638
	Correct predictions	51	48	
	Total predictions	103	76	
M2 ordinal	Accuracy	56%	62%	Chi-squared = 0.34724, df = 1, p-value = 0.556
	Correct predictions	58	47	
	Total predictions	103	76	
M3 ordinal	Accuracy	53%	65%	Chi-squared = 1.7722, df = 1, p-value = 0.1831
	Correct predictions	55	49	

	Total predictions	103	76	
M1 linear	Accuracy	33.98%	43.42%	Chi-squared = 1.278, df = 1, p-value = 0.2583
	Correct predictions	35	33	
	Total predictions	103	76	
M2 linear	Accuracy	36.89%	43.42%	Chi-squared = 0.5298, df = 1, p-value = 0.4667
	Correct predictions	38	33	
	Total predictions	103	76	
M3 linear	Accuracy	38.84%	46.05%	Chi-squared = 0.66284, df = 1, p-value = 0.416
	Correct predictions	40	35	
	Total predictions	103	76	
M1 ordinal 4 classes	Accuracy	45%	49%	Chi-squared = 0.22382, df = 1, p-value = 0.6361
	Correct predictions	46	38	
	Total predictions	103	77	
M2 ordinal 4 classes	Accuracy	41%	58%	Chi-squared = 4.8211, df = 1, p-value = 0.0281
	Correct predictions	42	45	
	Total predictions	103	77	
M3 ordinal 4 classes	Accuracy	47%	49%	Chi-squared = 0.045998, df = 1, p-value = 0.830
	Correct predictions	48	38	
	Total predictions	103	77	

Note. M1 = Model 1 contains the ten RFs, M2 = Model 2 contains age-14 distress, M3 = Model 3 contains both the RFs and age-14 distress as predictors for age-17 distress. Correct predictions = number of correctly predicted adolescents, Total predictions = number of adolescents that could have been predicted correctly, Accuracy = ratio correct predictions divided by total predictions. df = degrees of freedom.

Accuracy comparison for models including all RFs versus those including a subset of the RFs

**Model**	**Coefficient**	**All RFs**	**3 RFs**	**Proportion test summary**
M1 ordinal	Accuracy	63%	61%	Chi-squared = 0.047134, df = 1, p-value = 0.828
	Correct predictions	113	110	
	Total predictions	180	180	
M3 ordinal	Accuracy	66%	66%	Chi-squared = 0, df = 1, p-value = 1
	Correct predictions	118	118	
	Total predictions	180	180	
M1 linear	Accuracy	38.89%	37.78%	Chi-squared = 0.01175, df = 1, p-value = 0.914
	Correct predictions	70	68	
	Total predictions	180	180	
M3 linear	Accuracy	46.67%	44.44%	Chi-squared = 0.1008, df = 1, p-value = 0.7509
	Correct predictions	84	80	
	Total predictions	180	180	

Note. M1 = Model 1 contains the ten RFs, M2 = Model 2 contains age-14 distress, M3 = Model 3 contains both the RFs and age-14 distress as predictors for age-17 distress. Correct predictions = number of correctly predicted adolescents, Total predictions = number of adolescents that could have been predicted correctly, Accuracy = ratio correct predictions divided by total predictions. df = degrees of freedom.

Supplement VIII

For the CA+ group we tested four RFs in addition to gender, as those were significant in the multivariable model, namely: friendship support, family cohesion, brooding, and aggression. Those models were similarly predictive as the models with all 10 RFs and gender (change in accuracy: ordinal models from 45% to 50%; linear models from 43.01% to 35.48%). We also tested those two models while additionally including age-14 distress, which were again similar as the models with gender, the 10 RFs and age-14 distress (change in accuracy: ordinal models from 52% to 55%; linear models from 43.01% to 36.56%). Interestingly, while the accuracy of the ordinal models seems to increase with less RFs, the accuracy of the linear models seems to decrease.

For the CA- group we tested two RFs in addition to gender, as those were significant in the multivariable model, namely: negative self-esteem, and brooding. Those models were similarly predictive as the models with all 10 RFs and gender (change in accuracy: ordinal models from 67% to 64%; linear models from 41.18% to 43.53%). We also tested those two models while additionally including age-14 distress, which were again similar as (and potentially more accurate than) the models with gender, the 10 RFs and age-14 distress (change in accuracy: ordinal models from 59% to 62%; linear models from 43.53% to 50.59%).

For female adolescents we tested four RFs, as those were significant in the multivariable model, namely: friendship support, family cohesion, negative self-esteem, and brooding. Those models were similarly predictive as the models with all 10 RFs and CA (change in accuracy: ordinal models from 50% to 49%; linear models from 33.98% to 34.95%). We also tested those two models while additionally including age-14 distress, which were again similar as the models with the 10 RFs, age-14 distress and CA (change in accuracy: ordinal models from 53% to 53%; linear models from 38.84% to 38.84%).

For male adolescents we tested two RFs, as those were significant in the multivariable model, namely: negative self-esteem and brooding. Those models were similarly predictive as the models with all 10 RFs and CA (change in accuracy: ordinal models from 63% to 59%; linear models from 43.42% to 43.42%). We also tested those two models while additionally including age-14 distress, which were again similar as the models with the 10 RFs, age-14 distress, and CA (change in accuracy: ordinal models from 65% to 62%; linear models from 46.05% to 43.42%).

Here we did not test whether the accuracy differed significantly between the subgroups (i.e. CA+ vs CA-, and female vs male) as we tested the subgroups with different sets of RF predictors.

Supplement IX

Similar to the 3-class model, the 4-class model revealed a plausible distress severity solution, split in a low, low/moderate, moderate/high and a high distress severity class.

We also conducted three *ordinal* prediction models with the four-class distress variable as ordered categorical outcome variable. Of the three models one again contained the RFs (M1), one age-14 distress (M2) and one both (RFs and age-14 distress; M3) in addition to gender and CA. The three models had a low accuracy ranging from 46% to 53% (see Table below), resulting for all three models in about one in two adolescents who were predicted into their correct distress severity class. The results were comparable when we split the adolescents into CA+ (accuracy: M1 = 46%, M2 = 47%, M3 = 47%), CA- (accuracy: M1 = 52%, M2 = 57%, M3 = 56%), female (accuracy: M1 = 45%, M2 = 41%, M3 = 47%) and male groups (accuracy: M1 = 49%, M2 = 58%, M3 = 49%). Most of the prediction accuracies did not differ between the CA and gender subgroups (for details see Supplement VII); only model M2 revealed a significant effect for the gender subgroups ($Chi^2 = 4.821$, df = 1, $p = 0.028$).

Ordinal prediction analyses for the whole group: for RFs only (M1), age-14 distress only (M2), and RFs and age-14 distress together (M3)

	**M1: RFs only**		**M2: D14 only**		**M3: RFs & D14**	
	**observed**	**predicted**	**observed**	**predicted**	**observed**	**predicted**
Residual deviance	1193.96	-	1144.87	-	1131.00	-
ROC	-	low=0.74<br>l/m=0.61<br>m/h=0.74<br>high=0.74	-	low=0.80<br>l/m=0.62<br>m/h=0.79<br>high=0.80	-	low=0.80<br>l/m=0.63<br>m/h=0.79<br>high=0.78

Sensitivity	-	low=0.57<br>l/m=0.55<br>m/h=0.25<br>high=0.00	-	low=0.74<br>l/m=0.52<br>m/h=0.28<br>high=0.10	-	low=0.77<br>l/m=0.49<br>m/h=0.33<br>high=0.00
Specificity	-	low=0.75<br>l/m=0.54<br>m/h=0.89<br>high=1.00	-	low=0.78<br>l/m=0.64<br>m/h=0.86<br>high=1.00	-	low=0.76<br>l/m=0.67<br>m/h=0.86<br>high=1.00
Accuracy	-	0.46<br>low=0.66<br>l/m=0.55<br>m/h=0.57<br>high=0.50	-	0.52<br>low=0.76<br>l/m=0.58<br>m/h=0.57<br>high=0.55	-	0.53<br>low=0.76<br>l/m=0.58<br>m/h=0.60<br>high=0.50
Low distress severity	65	66 of which<br>- 37 correct<br>- 21 false l/m<br>- 07 false m/h<br>- 01 false high	65	73 of which<br>- 48 correct<br>- 19 false l/m<br>- 05 false m/h<br>- 01 false high	65	78 of which<br>- 50 correct<br>- 21 false l/m<br>- 06 false m/h<br>- 01 false high
Low/mod severity	65	89 of which<br>- 36 correct<br>- 27 false low<br>- 23 false h/m<br>- 03 false high	65	76 of which<br>- 34 correct<br>- 16 false low<br>- 24 false h/m<br>- 02 false high	65	70 of which<br>- 32 correct<br>- 15 false low<br>- 21 false h/m<br>- 02 false high
Mod/high severity	40	25 of which<br>- 10 correct<br>- 01 false low<br>- 08 false l/m<br>- 06 false high	40	30 of which<br>- 11 correct<br>- 01 false low<br>- 12 false l/m<br>- 06 false high	40	32 of which<br>- 13 correct<br>- 00 false low<br>- 12 false l/m<br>- 07 false high
High distress severity	10	0 of which<br>- 00 correct<br>- 00 false low<br>- 00 false l/m<br>- 00 false m/h	10	1 of which<br>- 01 correct<br>- 00 false low<br>- 00 false l/m<br>- 00 false m/h	10	0 of which<br>- 00 correct<br>- 00 false low<br>- 00 false l/m<br>- 00 false m/h

Note. D14 = age-14 distress. All models were computed with childhood adversity and gender as predictors. ROC = receiver operating characteristic. Accuracy = relative number of correctly predicted cases. Sensitivity = e.g. for low distress: the number of adolescents who are correctly predicted into the low distress group divided by all adolescent who are actually in the low distress group. Specificity = e.g. for low distress: the number of adolescents who are correctly not predicted into the low distress group divided by all adolescent who are actually not in the low distress group. Variable for which the proportional odds assumption was relaxed can be found in Supplement VI.

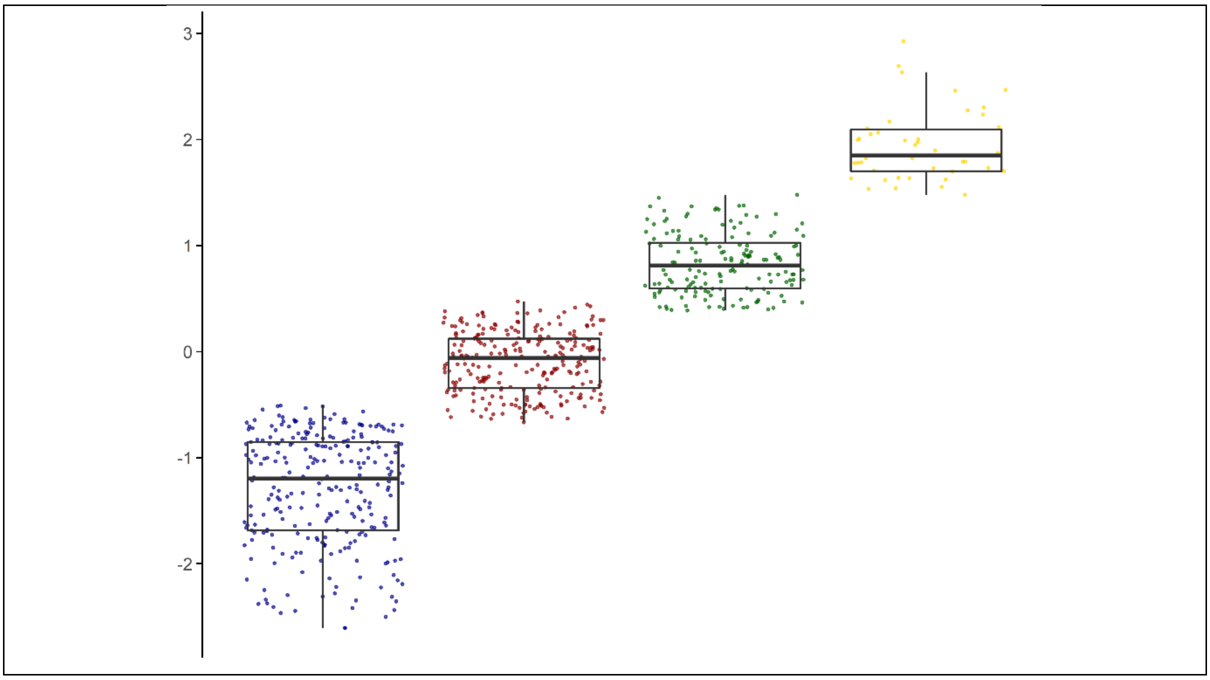

Figure 1. Four-class distress solution (low: n = 263; low/moderate: n = 260; moderate/high: n = 160; high: n = 42) plotted against the continuous distress severity scores. Center line = median (50% quantile); lower box limit =25% quantile; upper box limit = 75% quantile; lower whisker = smallest observation greater than or equal to the lower box limit -1.5 x Inter Quartile Range (IQR); upper whisker = largest observation less than or equal to the upper box limit + 1.5 x Inter Quartile Range (IQR).
